# Electron-Rich Acetylenic Nanostructures with *In Situ* Chelated Ultrafine Palladium Nanoclusters for Efficient Liquid-Phase Reaction Catalyst

**DOI:** 10.1021/acs.nanolett.6c02168

**Published:** 2026-08-10

**Authors:** Xinyu Su, Feng Yu, Houhong Song, Qiang Wang, Shengen Qiu, Zhibo Liu, Siyu Yao, Gaorong Han, Wencai Ren, Zongping Chen, Xinliang Feng

**Affiliations:** † State Key Laboratory of Silicon and Advanced Semiconductor Materials, School of Materials Science and Engineering, 12377 Zhejiang University, Hangzhou 310058, China; ‡ Faculty of Materials Science and Energy Engineering, 704566 Shenzhen University of Advanced Technology, Shenzhen 518107, China; § College of Chemical and Biological Engineering, Zhejiang University, Hangzhou 310058, China; ∥ Shenyang National Laboratory for Materials Science, Institute of Metal Research, Chinese Academy of Sciences, Shenyang 110016, China; ⊥ Faculty of Chemistry and Food Chemistry & Center for Advancing Electronics Dresden (CFAED), Dresden University of Technology, 01062 Dresden, Germany; # Max Planck Institute of Microstructure Physics, Weinberg 2, 06120 Halle, Germany

**Keywords:** ultrafine Pd nanoclusters, *in situ* deposition, acetylenic nanostructures, electron-rich environment

## Abstract

The development of uniformly distributed ultrafine metallic nanoclusters for advanced catalysts has attracted significant interest. However, synthesizing supports with specific electronic environments and well-defined pores to prevent aggregation remains challenging. Here, we report a facile palladium/copper cocatalyzed polycondensation to synthesize carbon-rich acetylenic nanostructures, poly­(1,3,5-tri­ethynyl­benzene) (PTEB), for the *in situ* chelation of ultrafine Pd nanoclusters (∼0.4 nm). The extensive π-conjugated configuration of PTEB provides an electron-rich environment that enhances Pd coordination through charge transfer, while its intrinsic pore structures ensure exceptional Pd dispersibility and effective spatial confinement. This synergistic electronic and spatial design in Pd/PTEB catalysts exhibits impressive catalytic activities in liquid-phase reactions, achieving a remarkable turnover frequency of 15 039 mol_C=C_ mol_Pd_^–1^ h^–1^ in the semihydrogenation of 1-octyne and a rate constant of 2.26 × 10^7^ min^–1^ g_Pd_^–1^ for 4-nitrophenol reduction. These performances represent a significant improvement over commercial and previously reported Pd catalysts, demonstrating promising advances for stable and active heterogeneous catalysts.

## Graphical abstract

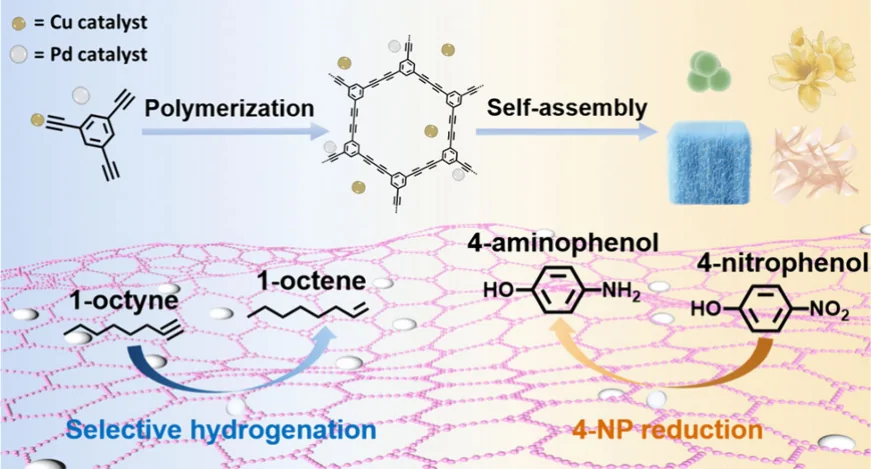

The development of advanced heterogeneous catalysts that incorporate uniformly distributed ultrafine metallic nanoclusters (UMNs) with meticulously controlled shapes has attracted considerable attention owing to their exceptional catalytic characteristics and diverse applications such as catalysis in chemical reaction, degradation, and photoreaction.^1–3^ Due to the high free energy of UMNs catalysts, tailoring the coordination environment is essential to ensure stability and electronic modulation of the metal sites.^4,5^ Despite the notable progress in fabricating UMN catalysts, maximizing UMN potential remains hindered by issues such as nanoparticle aggregation and weak metal–support interactions. Recently, carbon-rich conjugated polymers featuring diacetylene-linked benzene rings have emerged as a class of organic porous materials. These polymers uniquely combine π-conjugated frameworks with permanent nanopores,^6^ providing an ideal coordination environment with electron-rich cavities that effectively chelate transition metals.^7,8^ The extensive π-conjugation within these structures introduces additional electrons above the conjugated plane, enhancing the potential for robust charge transfer to metal atoms.^7^ Moreover, morphological modulation endows these catalysts with high surface areas and well-defined pore structures, confining the reaction space and thereby boosting the catalytic performance.^5^ Nevertheless, these conjugated polymers are typically obtained as amorphous particles with severe aggregation, thus limiting the dispersion of isolated metal sites and reducing the exposure of active sites.^6,9,10^ Therefore, the development of a facile synthesis approach for supportive materials with an optimal coordination environment and well-defined morphology is crucial. Such materials will ensure the *in situ* confinement of isolated metallic sites, maximizing the potential of UMN catalysts for outstanding activity and efficient atom utilization.

Herein, we report an efficient palladium/copper cocatalyzed polycondensation approach for the scalable synthesis of electron-rich acetylenic nanostructured poly­(1,3,5-tri­ethynyl­benzene) (PTEB) that forms well-defined shapes, ideal for the *in situ* chelation of ultrafine palladium (Pd) nanoclusters. By controlling the coupling rate, we can tailor the morphology evolving from nanoparticles (PTEB-NPs) to nanofibers (PTEB-NFs), nanoflowers, and nanosheets (PTEB-NSs). Density functional theory (DFT) calculations illustrate that the large π-conjugated configurations of the acetylenic PTEB nanostructures provide electron-rich coordination environments^7^ and robust charge transfer capabilities. Moreover, N_2_ adsorption–desorption characterizations confirm that the uniform pore distribution provides highly exposed active sites and reaction space confinement.^5^ Aberration correction transmission electron microscopy (AC-TEM) results further reveal the *in situ* chelation of highly dispersed ultrafine Pd nanoclusters (average size of ∼0.4 nm). This dual design of electronic and spatial pore structures of Pd/PTEB ensures impressive activity for various liquid-phase catalytic reactions. Notably, a remarkable turnover frequency (TOF) value of 15 039 mol_C=C_ mol_Pd_^–1^ h^–1^ toward 1-octyne semihydrogenation is achieved for the Pd/PTEB catalyst, which is superior to those of reported heterogeneous catalysts and more than 6 times higher than that of the commercial Lindlar catalyst (2335 mol_C=C_ mol_Pd_^–1^ h^–1^). Additionally, a high rate constant per unit mass of 2.26 × 10^7^ min^–1^ g_Pd_^–1^ was recorded for the reduction of 4-nitrophenol, outclassing the state-of-the-art Pd-based catalysts under similar reaction conditions (1.31 × 10^4^–7.3 × 10^6^).^11,12^

The palladium/copper cocatalyzed coupling of terminal alkynes under oxidative conditions is highly efficient for generating carbon-rich acetylenic frameworks.^13–18^ As illustrated in Figure 1 and Figure S1, the homocoupling of monomer 1,3,5-tri­ethynyl­benzene (TEB) is initiated by CuI/PdCl_2_(PPh_3_)_2_ catalysts using oxidizing agents such as O_2_, *p*-benzoquinone (PBQ), etc.^19^ The obtained PTEB nanostructures feature an extensive π-conjugation system linked by diacetylenic bonds with abundant electron-rich cavities. Figure 1a shows the divergent coupling pathways dictated by the catalyst loading. At a low Pd catalyst loading (0.5 mg, Figure 1c), the monomers are coupled to form a nanofiber morphology.^20^ Conversely, simultaneous multiple site coupling occurs at higher Pd loadings (such as 7.5 mg, Figure 1c), yielding nanoflower and nanosheet morphologies. It is reported that the formation of anisotropic nanostructures typically requires a higher chemical potential, which corresponds to an elevated concentration of feedstock.^21–23^ The chemical potential is proportional to the surface atom ratio of various morphologies (NSs > NFs > NPs). We speculate that the chemical potential of active species and the polymerization rate increase with the amount of Pd catalyst, driving the morphologic evolution from nanospheres to nanosheets (Figure 1b).^21,23^ Therefore, regulating the chemical potential via the catalyst concentration is the key innovation in this study for modulating PTEB nanostructures (details are provided in the Supporting Information). The topography statistics for tuning the polymerization condition (such as oxidation and catalyst amounts) are summarized in Figure 1c, and the corresponding scanning transmission electron microscopy (STEM) images of typical PTEB nanostructures obtained under different synthesis conditions are shown in Figure 1e and Figure S2. They demonstrate that the morphology evolved from NPs or NFs to nanoflowers and NSs with increased Pd catalytic loading (take O_2_ and PBQ, for example; Figure 1c). Figure 1d displays a digital photo of PTEB-NFs, which maintain a morphology of interconnected nanofibers.

**Figure 1:**
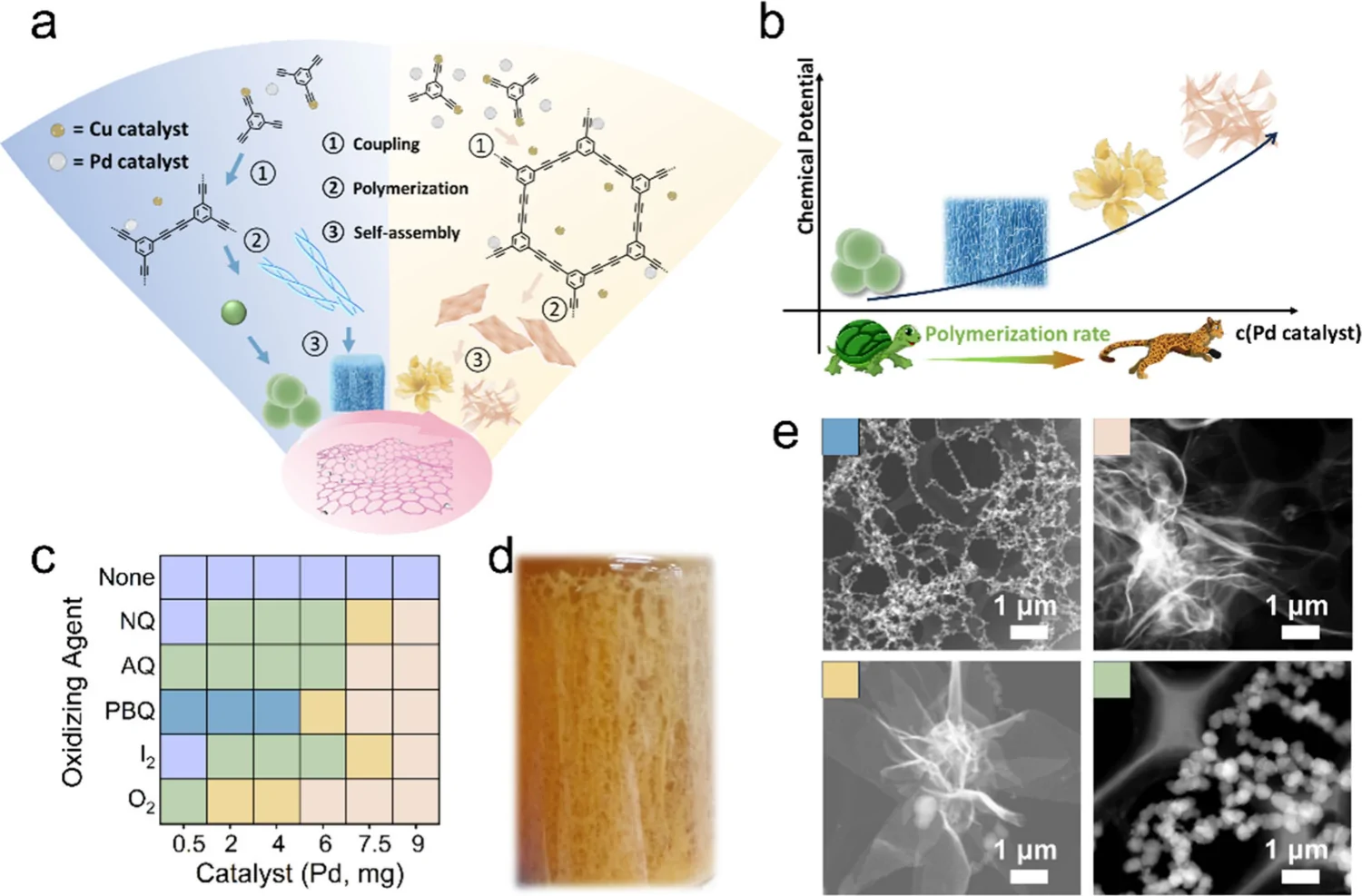
The morphology evolution mechanism of PTEB nanostructures. (a) Schematic illustration of the synthesis of PTEB with different morphologies. The dotted single bond indicates a continuation of the coupling. (b) Morphology evolution of PTEB according to the chemical potential of reaction systems with different concentrations of Pd catalyst. (c) Corresponding phase diagram of PTEB morphologies against the oxidants and Pd catalyst concentration. Color codes: blue (interconnected 1D NFs), green (NPs), yellow (nanosheet-assembled flowers), pink (stacked 2D NSs), and purple (failed synthesis). NQ, AQ, and PBQ represent naphthoquinone, anthraquinone, and *p*-benzoquinone, respectively. (d) Digital photo of obtained PTEB nanofibers. (e) STEM images of different PTEB morphologies, corresponding to different synthesis conditions shown in (c).

Nitrogen adsorption–desorption characterizations were performed to evaluate the pore structure of PTEB.^19^ For PTEB-NFs, the surface area is ca. 851 m^2^ g^–1^ based on Brunauer–​Emmett–Teller (BET) analysis (Figure S3), with the pore size distribution centered at about 0.6 nm (Figure S4), indicating a regular structure.^24–26^ In comparison, PTEB-NPs and PTEB-NSs exhibit a broader and more irregular pore distribution, resulting in their relatively smaller surface areas. A porous nanofiber structure is beneficial for liquid-phase catalysis. The uniform pores can provide a confined reaction space, while the interconnected nanofiber network helps reactants and products move through the catalyst more easily. The solid-state nuclear magnetic resonance (NMR) results in Figure 2a unequivocally determine the alkynyl-rich structure of PTEB. The sp^2^-hybridized sites C1 and C2 correspond to the peaks at 135.8 and 123.1 ppm, respectively, and the peaks at 81.4 and 76.4 ppm are ascribed to sp-hybridized sites C3 and C4.^24–27^ The overall chemical structure of the PTEB is further determined by X-ray photoelectron spectroscopy (XPS). As shown in Figure 2b, the C 1s spectrum of PTEB is deconvoluted into C≡C at 285.2 eV and C=C at 284.6 eV,^28,29^ with a ratio close to 1:1, consistent with the NMR results. The small ratio of C—O/C=O peaks likely stems from uncoupled terminal alkyne oxidation or adsorbed oxygen.^24–26^ The Fourier transform infrared spectroscopy (FTIR) spectrum (Figure S5) and Raman spectrum (Figure S6) of PTEB and the TEB monomer show the appearance of a peak at 2202 cm^–1^, characteristic of the —C≡C—C≡C— stretching vibration.^24–26^ The peaks near 1600 cm^–1^ are attributed to the skeletal vibrations of the aromatic ring. All these analyses reveal the formation of a conjugated polymer rich in benzene with diacetylenic linkages.

**Figure 2:**
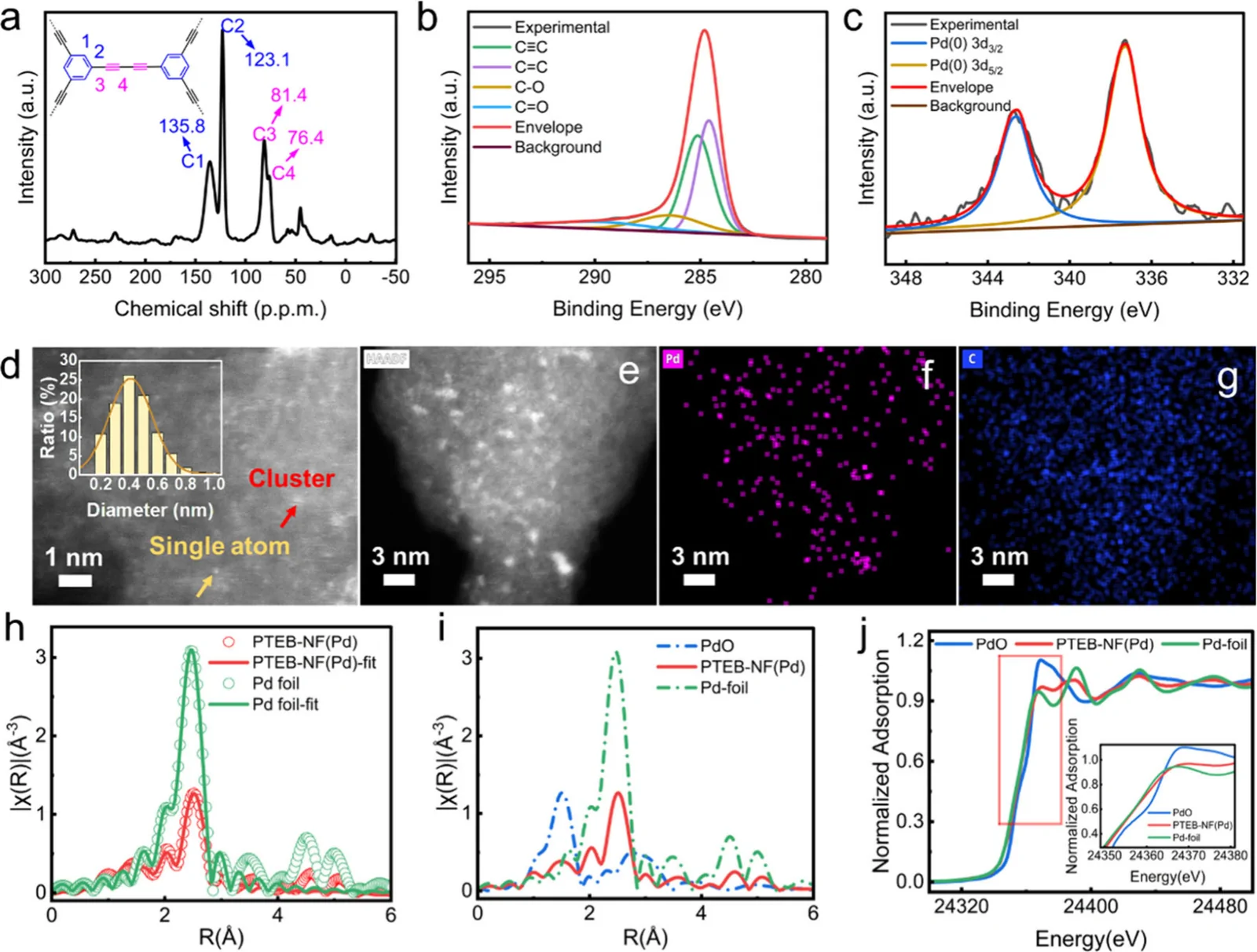
Structural characterization of PTEB and the chelate environment for Pd clusters. (a) ^13^C solid-state NMR spectrum of PTEB. (b) C 1s XPS spectrum and (c) Pd 3d XPS spectrum of PTEB. (d) AC-STEM images of PTEB (inset: statistical distribution of Pd cluster size in PTEB-NFs) and (e–g) HAADF-STEM and corresponding EDS mapping images of (f) Pd and (g) C elements. (h) Fitting curves of the FT spectra of PTEB-NFs and Pd foil using the ARTEMIS module of IFEFFIT. (i) FT-EXAFS spectrum of PTEB-NFs. (j) XANES profile of PTEB-NFs, with Pd foil and PdO as standard samples.

Pd has been widely used for liquid-phase catalytic reactions owing to its exceptional activity.^3,30^ The diverse forms and sizes of Pd active species have different performances in catalytic reactions.^31,32^ In this work, PdCl_2_(PPh_3_)_2_ serves as both the homocoupling catalyst and the source for the *in situ* deposition and chelation of Pd clusters onto the acetylenic bonds of PTEB. The actual Pd content in the final Pd/PTEB composites was quantitatively determined by ICP-OES, and the results are summarized in Table S1. Compared with the postdeposition process, *in situ* chelation generates ultrafine metallic nanostructures while effectively suppressing aggregation. Meanwhile, numerous Pd chelation reactions tend to occur owing to the electron-rich coordination environment in PTEB (including NFs, NPs, and NSs),^7,8^ achieving significant dispersions with more accessible metal active sites.

The high-resolution XPS core level spectrum of Pd 3d for Pd/PTEB are displayed in Figure 2c to characterize the valence of the *in situ* chelated Pd. The peaks at 337.2 and 342.7 eV correspond to 3d_5/2_ and 3d_3/2_ of Pd(0), and the binding energies are higher than those of standard metal Pd at 335.1 and 340.0 eV, indicating robust Pd–C interactions and charge transfer between the Pd clusters and the electron-rich PTEB support.^33,34^ To examine the possible residual Cu species introduced during the coupling reaction, Cu 2p XPS is further performed. No obvious Cu 2p signal was observed in the XPS spectrum of Pd/PTEB-NFs, indicating negligible Cu species on the catalyst (Figure S7). Both Pd nanoclusters and individual Pd single atoms are *in situ* chelated on PTEB as revealed by the AC-STEM image in Figure 2d. High-angle annular dark-field scanning transmission electron microscopy (HAADF-STEM, spherical aberration) and the corresponding elemental mapping confirm the uniform distribution of Pd throughout the PTEB framework. The size of Pd nanoclusters is primarily distributed in the range of 0.3–0.5 nm (with an average size of ∼0.4 nm; inset of Figure 2d). The successful chelation of uniform subnanometer Pd clusters demonstrates the superiority of *in situ* deposition for preventing metal aggregation (Figure 2d–f).

X-ray absorption fine structure (XAFS) spectroscopy was further conducted to study the coordination environments and electronic structure of Pd clusters in Pd/PTEB-NFs, along with Pd foil and PdO as reference samples. Figure 2h shows an excellent fit of the Fourier transformed (FT) spectra of Pd/PTEB-NFs and Pd foil using the ARTEMIS module of IFEFFIT^35^ with an average coordination number of 6.1 (±0.5) for Pd–Pd and 1.4 (±0.5) for Pd–C/O (Table S2). The FT extended X-ray absorption fine structure (FT-EXAFS) spectra of Pd/PTEB-NFs and reference samples in R space show an appreciable peak at 2.5 Å, corresponding to the shell Pd–Pd coordination. The weaker peak at 1.5 Å is ascribed to the first coordination shell of Pd in a bonding configuration of Pd–C or Pd–O (Figure 2i).^36^ The presence of oxidants in the synthetic system makes it impossible to completely exclude the existence of Pd–O. According to the X-ray absorption near edge structure (XANES) profiles (Figure 2j), the absorption edge of Pd/PTEB-NFs shifts scarcely to a higher energy. It is almost identical to that of Pd foil, indicating that Pd in Pd/PTEB-NFs is principally in a metallic state except for a slight oxidation state on the surface. The positive shift observed in XPS can be attributed to the surface sensitive electron deficiency of Pd species caused by Pd–C/O coordination and charge transfer with the PTEB support, while the XANES results reflect the averaged metallic Pd character of the Pd clusters. Additionally, the high-resolution transmission electron microscopy (HRTEM) image (Figure S8) and X-ray diffraction (XRD) pattern (Figure S9) also suggest the formation of highly dispersed atomic clusters instead of crystalline particles.^3,34^

Pd species are pivotal for facilitating the semihydrogenation of alkynes while suppressing overhydrogenation.^4,5^ Thus, a 1-octyne semihydrogenation test was used as a model reaction to demonstrate the efficiency of Pd/PTEB catalysts (Figure 3a). As shown in Figure 3b and Figure S10, Pd/PTEB-NFs exhibit the highest catalytic activity among the Pd/PTEB catalysts, achieving 99% conversion of 1-octyne within 20 min, which is higher than those of Pd/PTEB-NPs (80%) and Pd/PTEB-NSs (84%) under identical conditions. Impressively, the Pd/PTEB-NFs achieve a remarkably high TOF value of 15 039 mol_C=C_ mol_Pd_^–1^ h^–1^ with 98.50% selectivity of 1-octene, exceeding previously reported heterogeneous catalysts (Table S3);^11,37–39^ this TOF value is also more than 6 times higher than those of the commercial Lindlar catalyst (2335 mol_C=C_ mol_Pd_^–1^ h^–1^) and Pd loaded on graphene oxide (Pd/GO, 1765 mol_C=C_ mol_Pd_^–1^ h^–1^) (Figure 3c). These results demonstrate that the Pd/PTEB-NFs architecture significantly enhances the activity and selectivity for 1-octyne semihydrogenation.

**Figure 3:**
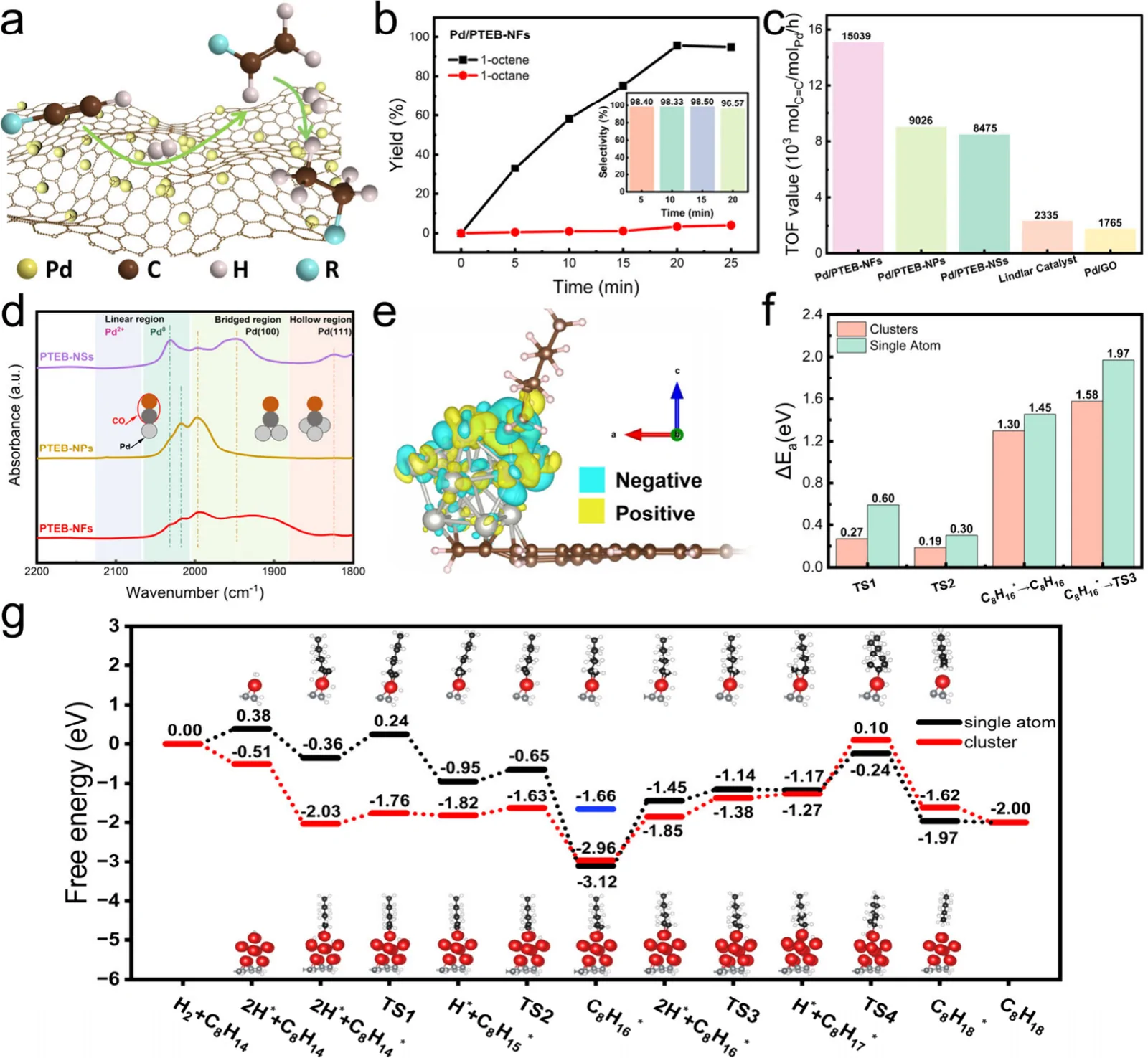
Catalytic performance and mechanistic investigation of Pd/PTEB for the selective hydrogenation of 1-octyne. (a) Schematic illustration for 1-octyne hydrogenation catalyzed by Pd/PTEB. R represents an alkyl chain without additional functional groups. (b) Plots of yield of 1-octene and 1-octane versus reaction time catalyzed by Pd/PTEB-NFs. Inset: the selectivity of 1-octene by using Pd/PTEB-NFs at different times. (c) TOF values of 1-octyne conversion catalyzed by different catalysts. (d) *In situ* CO-DRIFT spectra of Pd/PTEB-NFs, Pd/PTEB-NPs, and Pd/PTEB-NSs. (e) Differential charge density distributions of 1-octyne adsorbed on Pd cluster anchored PTEB. Yellow and cyan areas indicate electron accumulation and depletion, respectively. (f) Key energetic descriptors for 1-octyne semihydrogenation over Pd single atoms and Pd clusters. (g) Free energy profiles for 1-octyne hydrogenation on Pd clusters and Pd single atoms.

*In situ* CO-diffuse reflectance infrared Fourier transform spectroscopy (CO-DRIFT) was used to explore the surface coordination environment of Pd sites (Figure 3d). The signal of 2033 cm^–1^ is related to the linear adsorption of CO on metallic Pd sites.^40,41^ Compared with Pd/PTEB-NSs, the bands of Pd/PTEB-NPs and Pd/PTEB-NFs shift downward by almost 14 cm^–1^, indicating dipole coupling and a higher Pd electron density,^42^ which can benefit the catalytic performance. Bands at ∼1950 and 1995 cm^–1^ are assigned to bridge bonded CO on Pd, while the weak band centered at ∼1830 cm^–1^ is assigned to 3-fold hollow bonded CO on Pd.^42,43^ These characteristic CO adsorption modes reveal the coexistence of isolated Pd sites and small Pd clusters on the Pd/PTEB-NFs. To quantitatively compare the surface Pd structures, the ratio of nonlinear to linear adsorption of CO (R_ad_) was calculated from the integrated area of each spectral feature.^44,45^ The R_ad_ values of Pd/PTEB-NPs, Pd/PTEB-NSs, and Pd/PTEB-NFs are calculated to be 4.62, 5.44, and 13.95, respectively. The highest R_ad_ value of Pd/PTEB-NFs indicates a larger fraction of exposed Pd sites, which are beneficial for 1-octyne adsorption and activation. The underpotential deposition of Cu ions (Cu_upd_) was also used to measure the active surface area of Pd/PTEB. The Cu dissolution charge value (Q_Cu_) of Pd/PTEB-NFs (15.85 μC cm^–2^) is much greater than those of Pd/PTEB-NPs (6.72 μC cm^–2^) and Pd/PTEB-NSs (8.36 μC cm^–2^), indicating a larger number of surface-active sites (Figure S11).^46–48^

DFT calculations further reveal the electronic interaction between Pd/PTEB and 1-octyne.^49^ The thermodynamically most stable Pd/PTEB (models v and vii; both prefer to bind with the terminal alkynyl group) are displayed in Figures S12 and S13, and their differential charge density distributions are shown in Figure 3e and Figure S14. The significant charge transfer may be due to the electron-rich cavities in Pd/PTEB being an attractive coordination environment for Pd, leading to the strong interaction between the alkynyl groups and Pd.^7,8,21,23,50^ To further clarify the mechanistic origin of the high activity and selectivity, the stepwise hydrogenation pathways of 1-octyne on Pd clusters and Pd single atoms were calculated (Figure 3f,g). As shown in Figure 3g, 1-octyne adsorbs more strongly on Pd clusters, with an adsorption energy of −1.52 eV, than on Pd single atoms (−0.74 eV). The stronger interaction between 1-octyne and Pd clusters can better capture and activate 1-octyne before hydrogenation. Specifically, the energy barriers for TS1 and TS2 on Pd clusters are 0.27 and 0.19 eV, lower than those on single atoms (0.60 and 0.30 eV) (Figure 3f). These results indicate that Pd clusters act as more active sites than Pd single atoms for the initial activation and hydrogenation of 1-octyne to 1-octene. The high 1-octene selectivity originates from the competition between the product desorption and further hydrogenation. On Pd clusters, the desorption energy of 1-octene is 1.30 eV, lower than the barrier for the further hydrogenation to TS3 (1.58 eV), indicating that 1-octene preferentially desorbs rather than being overhydrogenated.^51^ Although Pd single atoms can also suppress overhydrogenation due to their higher hydrogenation barriers, 1-octene binds more strongly on Pd single atoms with a higher desorption energy of 1.45 eV. Therefore, Pd clusters provide a better balance between 1-octyne activation and 1-octene desorption, leading to higher activity and selectivity.

We further demonstrated the advantage of the Pd/PTEB catalyst in liquid-phase reactions using the reduction of 4-nitrophenol (4-NP) as another model reaction. The electron-rich PTEB framework facilitates the adsorption of electron-deficient nitro groups, inducing strong charge transfer and exceptional performance (Figure S15). In the presence of NaBH_4_, 4-NP is wholly reduced to 4-aminophenol (4-AP) within 30 s using Pd/PTEB (Figures S16 and S17). In contrast, catalysts prepared by the postdeposition of Pd on other carbon materials, including carbon black (Pd/C), multiwalled carbon nanotubes (Pd/MWCNT), and Pd/GO, exhibit significantly lower catalytic activities than Pd/PTEB, implying the superiority of the *in situ* confinement effect and the electronically favorable PTEB support. As shown in Figure 4a, the Pd/PTEB-NFs catalyst exhibits a notable improvement in the rate constant (k) with a value of 7.9 min^–1^, where k is determined from the slope of the pseudo-first-order kinetic plot of ln­(C_t_/C_0_) versus reaction time. This value is 3.3 and 3.8 times higher than those of Pd/PTEB-NSs and Pd/PTEB-NPs, respectively. The superior kinetics can be attributed to the uniform porous nanofiber architecture of Pd/PTEB-NFs, which helps stability, highly disperses Pd species, shortens diffusion pathways, facilitates the mass transfer of 4-NP, NaBH_4_, and 4-AP, and increases the accessibility of Pd active sites (Figures S3 and S4). As a result, the reduced mass transfer resistance and enhanced reactant active site contact lead to a high mass-normalized rate constant k′ of 2.26 × 10^7^ min^–1^ g_Pd_^–1^, which surpasses those of previously reported palladium-based catalysts at room temperature (Table S4).^3,11,34,50,52^

**Figure 4:**
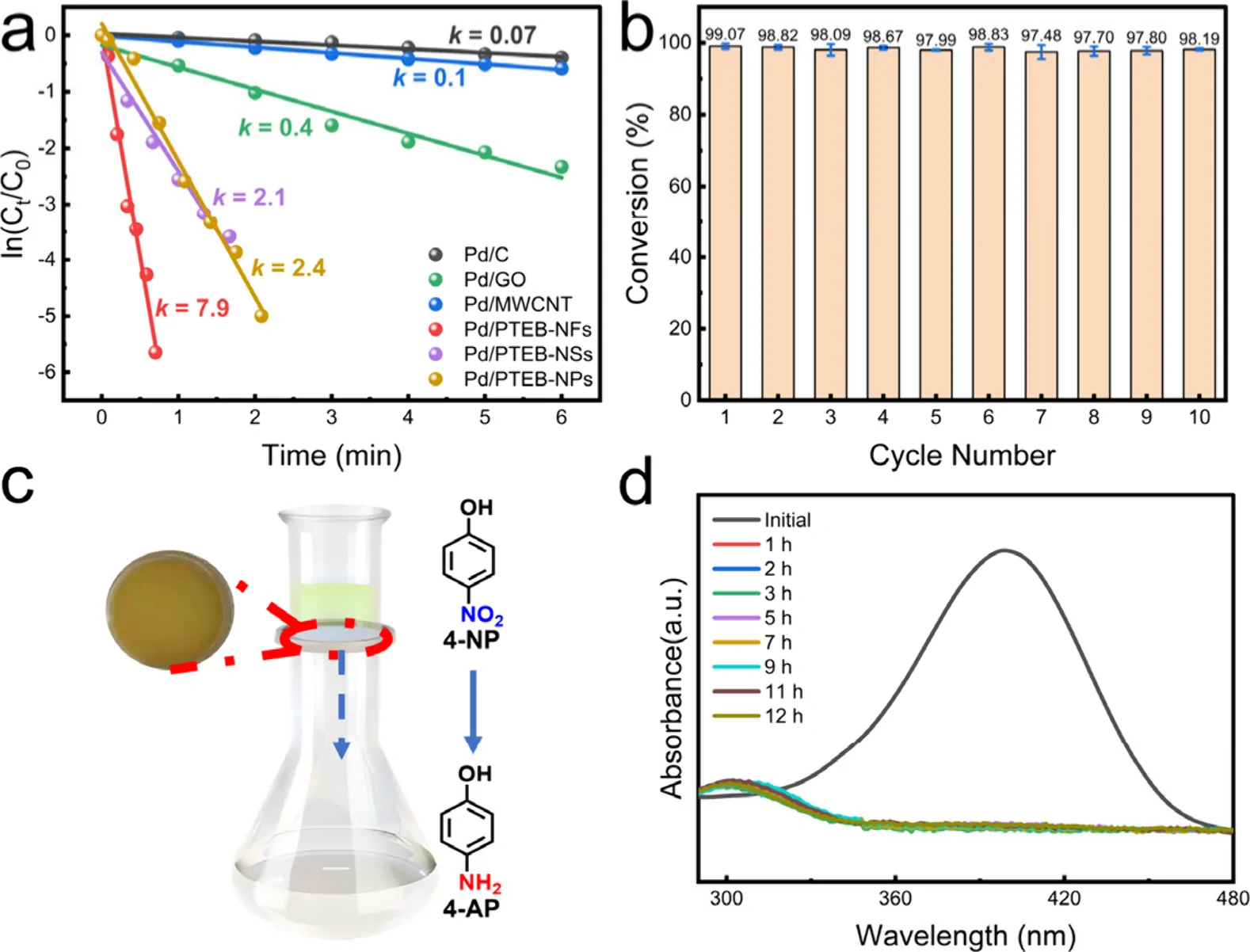
Catalytic performance and recyclability of Pd/PTEB for 4-nitrophenol reduction. (a) Plots of ln­(C_t_/C_0_) as a function of time for the reduction of 4-NP catalyzed by different catalysts. (b) 4-NP catalytic reduction performance of Pd/PTEB over ten cycles. (c) Schematic illustration to evaluate the performance of Pd/PTEB in 4-NP reduction of continuous flow. (d) Time-dependent UV–vis absorption spectra during the catalytic reduction of 4-NP.

Pd/PTEB-NFs also showed excellent catalytic stability. As shown in Figure 4b, the catalyst maintained 98% conversion after ten cycles without Pd aggregation (Figure S18) and remained stable for 12 h in continuous-flow tests (Figure 4c,d and Figure S19). Finally, the Pd/PTEB can also be used for the reduction of other nitroarene derivatives and dyes (Figure S17), representing remarkable catalytic performances and significantly enhanced rate constants regardless of the type or position of the substituents (Figures S20 and S21).

Through these comprehensive studies, we infer that the catalytic performance of Pd/PTEB-NFs originates from the integrated electronic and spatial regulation of Pd active sites. The electron-rich π-conjugated frameworks promote charge transfer to the chelated Pd sites,^3,21,23^ while the coordination environment stabilizes ultrafine Pd nanoclusters and suppresses aggregation.^7^ In parallel, the permanent nanopores confine the reaction space and expose accessible Pd sites, thereby improving Pd atom utilization and catalytic durability. The dual design of the electron-rich coordination environment and porous nanostructure is central to the high activity of Pd/PTEB-NFs in liquid-phase catalysis.

In summary, we have successfully constructed an electron-rich acetylenic PTEB nanostructure for the *in situ* chelation of uniformly distributed ultrafine Pd nanoclusters. Notably, the Pd/PTEB-NFs catalyst exhibits impressive catalytic activities toward a series of liquid-phase reactions, achieving a remarkable TOF value of 15 039 mol_C=C_ mol_Pd_^–1^ h^–1^ for 1-octyne semihydrogenation and a rate constant k′ value of 2.26 × 10^7^ min^–1^ g_Pd_^–1^ for 4-NP reduction, outperforming previously reported Pd-based heterogeneous catalysts and commercial catalysts. This outstanding catalytic performance is due to the substantial charge transfer from the electron-rich coordination environment of PTEB with well-defined porous architectures, which leads to high Pd atom utilization and confinement of the reaction space. These discoveries provide a new perspective to the catalysis field through the rational design of complex and well-defined electron-rich conjugated nanostructures for the chelation of isolated metal sites with advanced catalytic activity.

## Supplementary Material



## Notes

Experimental details, materials, and methods; supplemental SEM, AC-STEM, HRTEM, and XRD data; N_2_ adsorption–desorption isotherms and pore size analysis; FTIR and Raman spectra; electrochemical Cu_upd_ analysis; DFT models and charge density analyses; and additional catalytic data, kinetics, and comparison tables (PDF)
