# Supplementary material for: Electron-Rich Acetylenic Nanostructures with In Situ Chelated Ultrafine Palladium Nanoclusters for Efficient Liquid-Phase Reaction Catalyst
Source: Nano Lett. 2026 Aug 10;26(32):10707–14. doi: 10.1021/acs.nanolett.6c02168 (PMC13495940; doi:10.1021/acs.nanolett.6c02168)
Supplement: Supplementary file 1 [file nl6c02168_si_001.pdf]

Electron-rich acetylenic nanostructures with *in situ* chelated ultrafine palladium nanoclusters for efficient liquid-phase reaction catalyst

Supporting Information

Xinyu Su [a§], Feng Yu [a§], Houhong Song [c], Qiang Wang [d], Shengen Qiu [a], Zhibo Liu [d], Siyu Yao [c], Gaorong Han [a], Wencai Ren [d], Zongping Chen [b*], Xinliang Feng [e,f*]

[a] State Key Laboratory of Silicon and Advanced Semiconductor Materials, School of Materials Science and Engineering, Zhejiang University, Hangzhou 310058, China

[b] Faculty of Materials Science and Energy Engineering, Shenzhen University of Advanced Technology, Shenzhen 518107, China

[c] College of Chemical and Biological Engineering, Zhejiang University, Hangzhou 310058, China

[d] Shenyang National Laboratory for Materials Science, Institute of Metal Research, Chinese Academy of Sciences, Shenyang 110016, China

[e] Faculty of Chemistry and Food Chemistry & Center for Advancing Electronics Dresden (CFAED), Dresden University of Technology, 01062 Dresden, Germany

[f] Max Planck Institute of Microstructure Physics, Weinberg 2, 06120 Halle, Germany

§ Xinyu Su and Feng Yu contributed equally to this work.

*Email: chenzongping@suat-sz.edu.cn， xinliang.feng@tu-dresden.de

Experimental section

Synthesis of PTEB-NFs. In typical synthesis of PTEB, 5 mg TEB, 4.8 mg p-benzoquinone (PBQ), 0.5 mg $PdCl_2(PPh_3)_2$, and 1 mg CuI were dispersed into 10 mL triethylamine and 10 mL THF under Ar atmosphere. The solution was heated at 60°C for 48 h to develop the interconnect network product and then cooled down to room temperature. For the comparison of the addition of different doses of palladium catalyst, only 2, 4, 6, 7.5 and 9 mg $PdCl_2(PPh_3)_2$ was changed.

Synthesis of PTEB-NSs. 5 mg TEB, 7.5 mg $PdCl_2(PPh_3)_2$, and 2 mg CuI were dispersed into 10 mL triethylamine and 10 mL THF under atmosphere.The solution was heated at 60°C for 1 h to develop the interconnect network product and then cooled down to room temperature.

Synthesis of PTEB-NPs. 5 mg TEB, 0.5 mg $PdCl_2(PPh_3)_2$, and 1 mg CuI were dispersed into 10 mL triethylamine and 10 mL THF under atmosphere. The solution was heated at 60°C for 48 h to develop the interconnect network product and then cooled down to room temperature.

Synthesis of Pd/PTEB. The synthesized Pd/PTEB was cleaned with acetone, ethanol and methanol in turn, and finally dissolved in methanol solution, followed by ultrasonic dispersion with a cell shredder, and then the distributed PTEB-NFs methanol solution was mixed with a prepared sodium borohydride solution, and the infiltration reduction was carried out in a water bath at 30°C.

Synthesis of I_2-serial PTEB. In typical synthesis of I_2-PTEB, 5 mg TEB, 11.3 mg I_2

(equivalent to PBQ), 0.5 mg $PdCl_2(PPh_3)_2$, and 1 mg CuI were dispersed into 10 mL triethylamine and 10 mL THF under Ar atmosphere. The solution was heated at 60°C for 48 h to develop the interconnect network product and then cooled down to room temperature. For the comparison of the addition of different doses of palladium catalyst, only 2, 4, 6, 7.5 and 9 mg $PdCl_2(PPh_3)_2$ was changed.

Synthesis of O_2-serial PTEB. In typical synthesis of O_2-PTEB, 5 mg TEB, 0.5 mg $PdCl_2(PPh_3)_2$, and 1 mg CuI were dispersed into 10 mL triethylamine and 10 mL THF under air atmosphere. The solution was heated at 60 °C for 48 h to develop the interconnect network product and then cooled down to room temperature. For the comparison of the addition of different doses of palladium catalyst, only 2,4,6,7.5 and 9 mg $PdCl_2(PPh_3)_2$ was changed.

Synthesis of anthraquinone (AQ)-serial PTEB. In typical synthesis of AQ-PTEB, 5 mg TEB, 9.3 mg AQ (equivalent to PBQ), 0.5 mg $PdCl_2(PPh_3)_2$, and 1 mg CuI were dispersed into 10 mL triethylamine and 10 mL THF under Ar atmosphere. The solution was heated at 60 °C for 48 h to develop the interconnect network product and then cooled down to room temperature. For the comparison of the addition of different doses of palladium catalyst, only 2, 4, 6, 7.5 and 9 mg $PdCl_2(PPh_3)_2$ was changed.

Synthesis of naphthoquinone (NQ)-serial PTEB. In typical synthesis of NQ-PTEB, 5 mg TEB, 7.02 mg NQ (equivalent to PBQ), 0.5 mg $PdCl_2(PPh_3)_2$, and 1 mg CuI were dispersed into 10 mL triethylamine and 10 mL THF under Ar atmosphere. The solution was heated at 60°C for 48 h to develop the interconnect network product and then

cooled down to room temperature. For the comparison of the addition of different doses of palladium catalyst, only 2, 4, 6, 7.5 and 9 mg $PdCl_2(PPh_3)_2$ was changed.

Catalytic reduction of nitroarenes. The aqueous solution of $NaBH_4$ (1.30 mL, 0.2 M) and the corresponding nitroarenes (0.10 mL, 1 mM) were mixed with deionized water (1.60 mL). The mixed solution was stirred 5 min under room temperature and then the homogeneous dispersion of PTEB-NFs and PTEB-NPs (50 μL, 1.0 mg mL^{-1}), PTEB-NSs, Pd/MWCNT, Pd/GO, and Pd/C was added (30 μL, 1.0 mg mL^{-1}), respectively. The catalytic reduction reaction was monitored by UV-Vis spectra at short intervals in the range 240-550 nm. For the cycling test, the catalyst was collected by filtration after each 4-NP reduction run, washed with water and ethanol, and then used for the next run with fresh 4-NP and NaBH4 solutions. This procedure was repeated for ten cycles. In the case of continuous flow reaction model, the catalyst was packed into the reactor, and the pump power was precisely regulated to stabilize the flow rate of the mixed solution of 4-nitrophenol and $NaBH_4$ at 8 mL/min. The filtrate was collected at regular intervals for UV-Vis detection.

Catalytic degradation of organic dyes. The methyl orange (0.08 mL, 0.05 mmol L^{-1}), $NaBH_4$ (1.05 mL) were mixed with 1.87 mL deionized water, the mixture was stirred 5 min under room temperature. And then, the suspension of PTEB-NFs (50 μL, 1.0 mg mL^{-1}) was added into the solution, the catalytic degradation reaction was monitored by UV-Vis in the range 300-600 nm. Similarly, the experimental conditions of catalytic degradation of Congo red are the same as methyl orange, UV- Vis spectra range spans from 650 to 275 nm.

Semi-hydrogenation of 1-octyne. This catalytic reaction was carried out at 75 ℃ in oil bath and under static hydrogen atmosphere (1 bar). In a typical experiment, the ethanol (10 mL), ethanolic suspension of PTEB-NFs (6 mg, Pd: 0.39 μmol) or PTEB-NSs (1 mg, Pd: 0.66 μmol) or PTEB-NPs (10 mg, Pd: 0.50 μmol), 1-octyne (73.5 μL, 0.5mmol), and n-decane (10 μL, 0.05 mmol) were successively added into a glass reactor. Here, n-decane was used as internal standard. The residual air in the reactor was expelled by flushing with hydrogen for several times before the glass reactor was sealed. The pressure in the reactor was maintained at normal atmospheric pressure. Then the mixture was stirred at 600 rpm using a magnetic bar during the catalytic reaction. Small portion of the solution was taken out at different time intervals and analyzed with a gas chromatography.

Materials and Methods. 1,3,5-triethynylbenzene (TEB) was pretreated with liquid column chromatography for purification before synthesis. All the rest solvents and reagents are commercially available and used without further purification, unless otherwise noted.

Characterization. All the SEM images were performed using a JEOL JSM-IT800 microscope. TEM, HRTEM and STEM images and EDS mapping analyses were recorded on a JEOL F200 microscope at 200 kV and FEI Titan ChemiSTEM equipped with an image-side spherical aberration corrector at 200 kV. The samples were deposited on TEM grids from alcohol dispersion after sonication for 2 min. The UV-Vis absorption spectra were recorded using a Cary5000 UV-Vis NIR spectrophotometer from Agilent Technologies, Inc., with a scanning speed of 2000

nm/min. The crystal structure of the samples was investigated by X-ray diffractometer (XRD) using a Rigaku Smartlab 3kW diffractometer with Cu Kα irradiation (λ = 1.54 Å). All surface chemical state and composition of the as-synthesized samples were characterized by XPS with monochromatic Al Kα (1486.69 eV) X-ray radiation using an X-ray photoelectron spectroscopy instrument (Thermo Scientific ESCALAB 250Xi). Raman spectra were measured with a WITec alpha 300 R Raman spectrometer excited at 532 nm. The Fourier transform infrared spectroscopy (FTIR) was acquired on a Thermo Scientific Nicolet iS20 spectrometer. The thermogravimetric analysis (TGA) curves were collected by a Mettler Toledo TGA/DSC3+ instrument with a heating rate of 10 °C/min. Nitrogen adsorption/desorption measurements were performed at 77 K using Quantachrome Autosorb IQ gas-sorption system.

X-ray absorption fine structure (XAFS) was performed with Si (311) crystal monochromators at the beamline BL14W1 at the Shanghai Synchrotron Radiation Facility (SSRF) (Shanghai, China). Before the analysis at the beamline, samples were pressed into thin sheets with 1 cm in diameter and sealed using Kapton tape film with Pd foil and PdO measured in transmission mode as standards, using a 4-channel Silicon Drift Detector (SDD) Bruker 5040 at room temperature. Pd *K*-edge extended X-ray absorption fine structure (EXAFS) spectra were recorded in transmission mode. Negligible changes in the line-shape and peak position of Pd *K*-edge XANES spectra were observed between two scans taken for a specific sample. The data pretreatment and fitting of EXAFS spectra were performed using IFEFFIT[1] packages of Demeter. The EXAFS oscillation was fitted according to back-scattering equation, using FEFF

models generated from the crystal structures of Pd (Fm3m). The measured Pd foil data were fitted as the standard for amplitude reduction factor S02.

More detail for solid-state NMR spectroscopy measurement: Magic angle spinning (MAS) experiments were conducted using 3.2 mm double-resonance probes at a spinning rate of 15kHz. The spectra were collected at Larmor frequencies of 600 MHz for ^{1}H, 150.8 MHz for ^{13}C. The 1H spectra are referenced to Adamantane at 1.85 ppm, ^{13}C spectra are referenced to Adamantane at 38.48 ppm. ^{1}H spectra were collected via direct excitation at the radio frequency field (RF) of 100 kHz, with a relaxation delay time of 2 s. Cross Polarization (CP) was used to acquire ^{13}C spectra, with a contact time of 3 ms and relaxation time of 2 s. The liquid NMR spectra of 1,3,5-Triethynylbenzene (TEB) were measured using Bruker Avance-400 at 400 MHz.

Formation mechanism. To uncover the origins of morphology evolution of PTEB nanostructures, we further summarize the various PTEB morphologies synthesized under different conditions, as shown in Figure 1c. It is obvious that the growth of nanosheet structure of PTEB appears on relatively high $PdCl_2(PPh_3)_2$ catalyst concentrations and rapid polymerization rate. Taking O_2 as oxidant for example, the morphology of PTEB evolves from PTEB-NPs to PTEB-NSs as the amount of Pd catalyst increases (Figure 1c). Besides, the usage of other oxidants results in nearly the same law of morphology evolution (Figures S22-S27). Of note, a low Pd catalyst concentration and the use of PBQ as oxidant promote the formation of PTEB-NFs.

To elucidate the underlying principles and monitor the growth process of the well-defined PTEB nanostructures, we propose a mechanism according to a chemical

potential-controlled crystal growth model reported previously.[2-7] It is reported that the formation of anisotropic nanostructures necessitates a solution with a higher chemical potential, which corresponds to an elevated concentration.[2, 8-9] Accordingly, Figure 1b presents a reasonable relationship between the morphology evolution of PTEB nanostructures and the chemical potential of active particles in the system by adjusting the Pd-catalyst concentration. Consequently, a high concentration of $PdCl_2(PPh_3)_2$catalyst impacts the formation of active intermediate precursor Pd-TEB (see details in Figure S1), resulting in an increased number of nuclei and thereby enhancing the chemical potential within the system.[10] The chemical potential is proportional to the surface atom ratio of various morphologies (NSs>NFs>NPs) deduced from Gibbs-Thompson equation (detailed in Supporting Information, Equation (S1)–(S7) and Figure S28).[7, 11] Thereby, the TEB units surrounding the large number of nucleation sites grow in the form of nanosheet morphology (right part of Figure 1a) to reach the thermodynamic steady state.[12-14] In contrast, the morphology of PTEB tends to evolve into nanospheres at low concentrations of Pd catalyst attributed to the convergence of growth rate in different directions (left part in Figure 1a).[7] Therefore, we can speculate that the chemical potential of active species and the polymerization rate increase with the amount of Pd catalyst, which causes the morphologic evolution from nanospheres to nanosheets (Figure 1b).

In view of the appearance of NFs morphology, it can be attributed to the oxidation capacity of different oxidants and their difference of solubility in the system. Stronger oxidizing oxidants lead to a higher concentration of active substances (Pd-TEB, step 4

and 2 in Figure S1,) involved in the homocoupling cycle.[15-16] Correspondingly, a relatively higher chemical potential is initiated and thus provides a suitable energy for elongated growth in one direction. Specifically, the solubility of PBQ in THF solvent exceeds that of O_2, resulting in a relatively higher chemical potential for Pd-TEB in low Pd concentration system compared to the case using O_2. This difference accounts for the 1D nanofiber growth in the PBQ system, while it is not present in O_2 system. Consequently, the aforementioned discussion outlines the morphology evolution mechanism through the control of chemical potential by regulating the catalysts and oxidants of the homocoupling reaction.

Details analysis of Gibbs−Thompson equation. The Gibbs−Thompson equation is the basis of the classic crystallization theory which could provide a starting point for us (S1).

$$S_r = S_b \exp(2\sigma V_m / rRT) \qquad \text{(S1)}$$

Where r is the radius of the crystal, σ is the specific surface energy, V_m is the molar volume of the material, S_b and S_r are the solubility of bulk crystal and crystals with a radius r, R is the gas constant, and T is the absolute temperature.

The Gibbs-Thompson equation tells us that the solubility of a given sized crystal is strongly dependent on its size. However, the solubility of a crystal with its size in nanometer regime is a relative term, since it is impossible to reach an ultimate chemical equilibrium between the nanocrystal and the monomers at the concentration S_r

determined by S1. This is so because nanocrystals are thermodynamically mesostable species. Therefore, it should be more thermodynamically relevant to consider S_r as being a measure of the chemical potential of nanocrystals. Therefore, the chemical meaning of the Gibbs-Thompson equation is that the chemical potential of a collection of nanocrystals with a given radius r is the same as that of the monomers at the concentration S_r.

A simple mathematic treatment can change S1 to S2:

$$RTlnS_r = RTlnS_b + 2\sigma V_m/rRT \quad \text{(S2)}$$

When μ_r and μ_b, represent the chemical potentials of the crystals with a radius r and with an infinite size, we can obtain S3 from S2. Here, crystals with different sizes share the same standard state is assumed.

$$\mu_r = \mu_b + 2\sigma V_m/rRT \quad \text{(S3)}$$

For spherical (particle) morphology, the number of the surface atoms and the total atoms should be proportional to the surface area and the volume, respectively. If the surface atom ratio is defined as δ_p, then

$$\delta_p = (k_1 4\pi r^2)/(k_2 4\pi r^3/3) = k_3\ 1/r \quad \text{(S4)}$$

Which k_1, k_2 and k_3 are all proportional constants.

Combining S3 and S4 and setting the chemical potential of infinite sized crystals as the relative standard, the relative chemical potential of a crystal with a finite size should then be proportional to the surface atom ratio, δ.

$$\mu_r \propto \delta \quad \text{(S5)}$$

S5 indicates that the relative chemical potential of crystals is simple proportional to their surface atom ratio. In principle, the multidimensional growth occurs when the chemical potential of the active precursors in the solution is much higher to evolute an anisotropic shape for diffusion-controlled growth.[2, 7]

For fiber morphology, the volume of fiber is set to a constant value, if the aspect ratio is defined as *x*, then

$$\delta_f = k_4\, x^{-1/3}(2x+1) \quad \text{(S6)}$$

For sheet morphology, the volume of fiber is also set to a constant value and the thickness of nanosheet is *d*, then

$$\delta_s = k_5\, 1/d \quad \text{(S7)}$$

For instance, the volume of all the nanostructures in Figure S28 is set to a constant value. That is, when the nanostructure is elongated from a dot to a rod or a thin rectangle with its surface atom ratio increase by a factor of ca. 3 or 104 respectively, and the relative chemical potential needed also increases.

Details of density functional calculations. All density functional calculations are performed using the projected augmented wave method implemented in the Vienna Ab-Initio Simulation Package (VASP) code.[17] The plane-wave basis cutoff is set to 400 eV. The Perdew, Burke, and Ernzerhof (PBE) functional (which is considered a reliable function in organic and conjugated system) is utilized in structural optimizations and

the B3LYP functional with D3 correction (which is commonly used to calculate the weakly interacting system such as adsorption energy) is used to assess the adsorption energies.[18-19] All structure optimizations are performed until the change of the total energy is smaller than 1×10^{-4} eV between two ionic steps. For modelling the terminal alkyne, a=58 Å, b≈31 Å, c=30 Å cells with periodicity along only b orientation were utilized, with a Gamma-centered 1×1×1 K-points sampling. Further expanding the cell along a or c orientations yield the same results in the test calculations. All structures are visualized using VESTA.[20] For Pd clusters, the cluster structure and atom are designed according to the value of its average coordination number (Table S2).

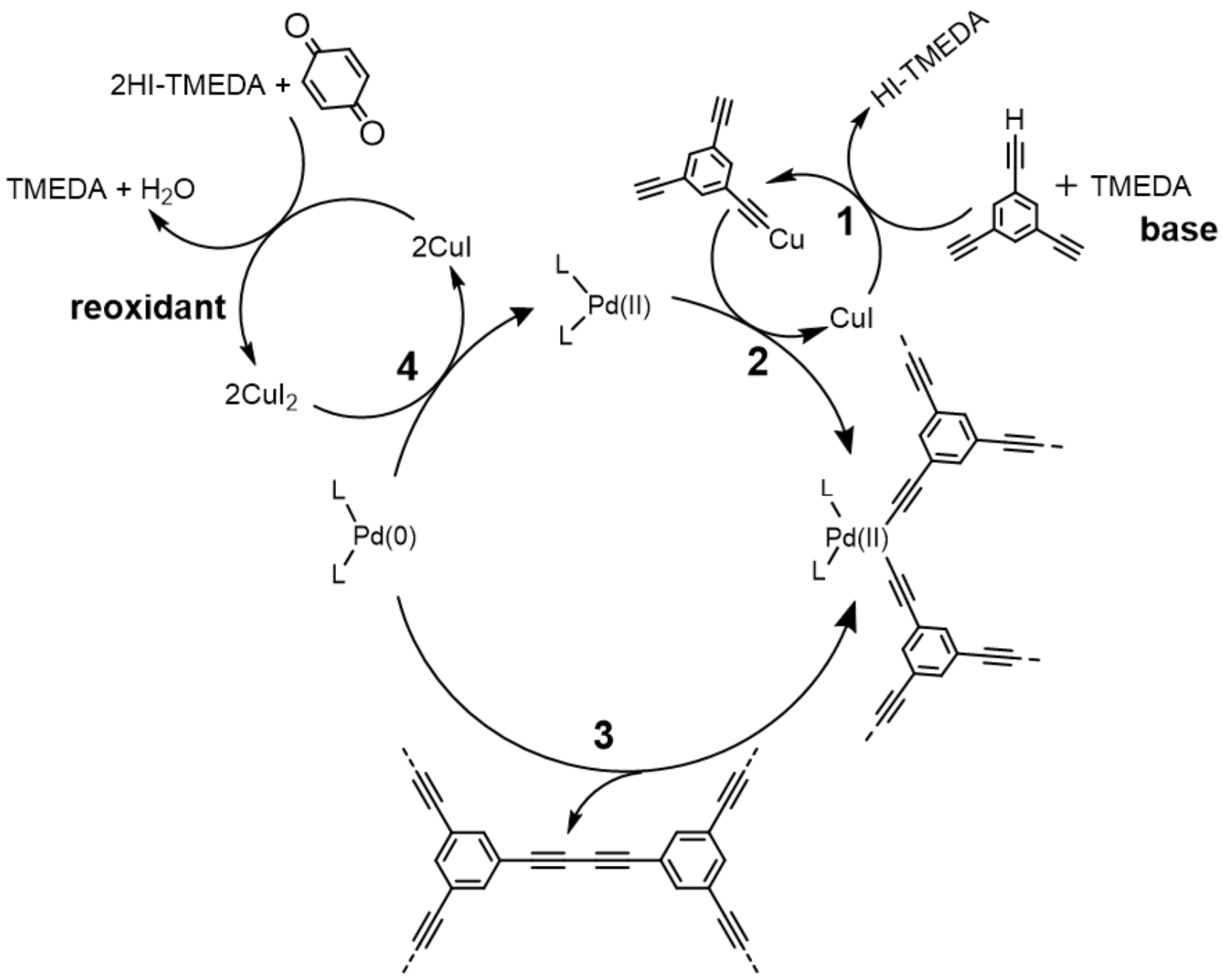

Figure S1. Diagram of the coupling of alkynyl groups and catalyst in reaction cycle, using PBQ as an example.

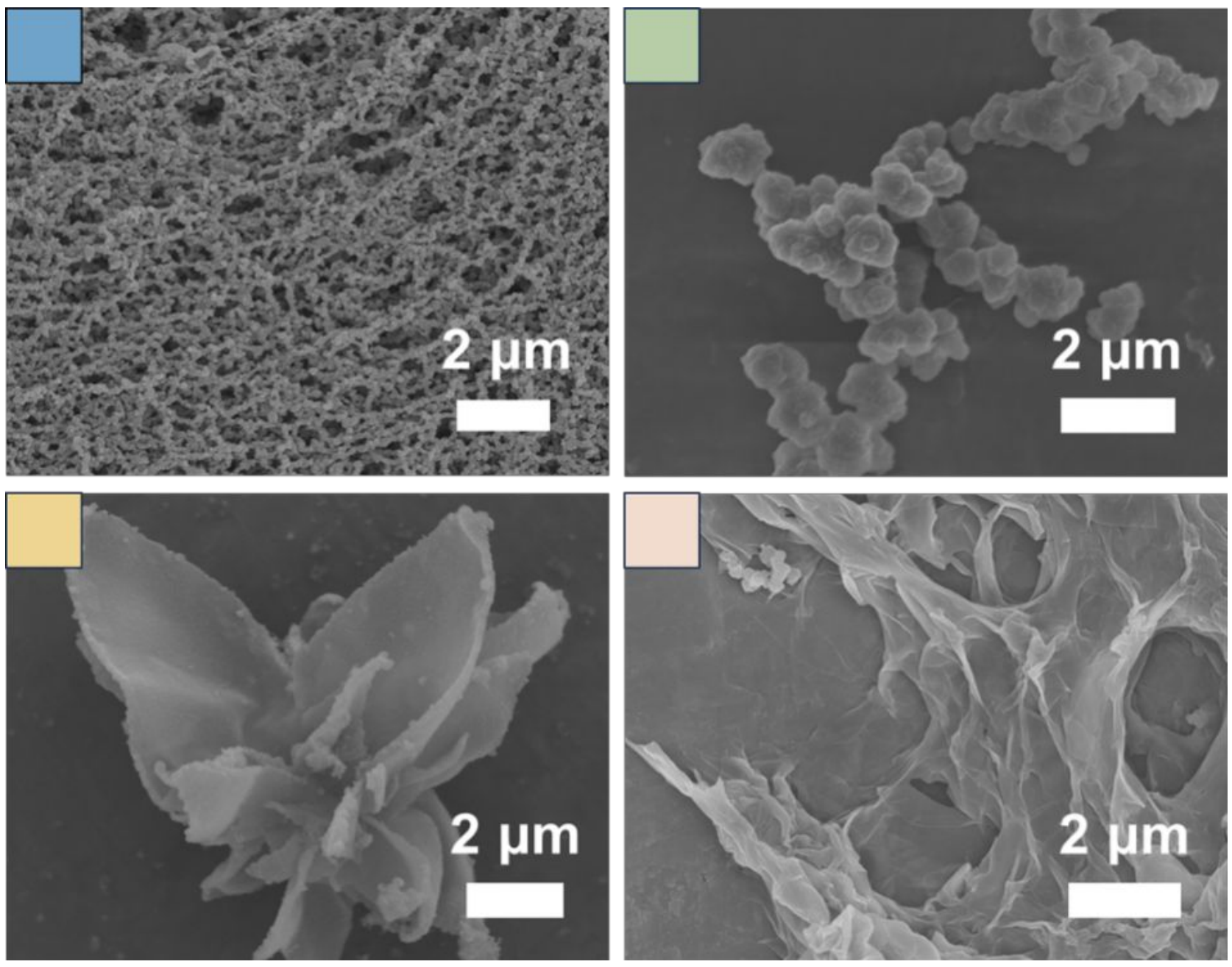

Figure S2. SEM images of different PTEB morphologies, corresponding to different synthesis conditions shown in Figure 1c.

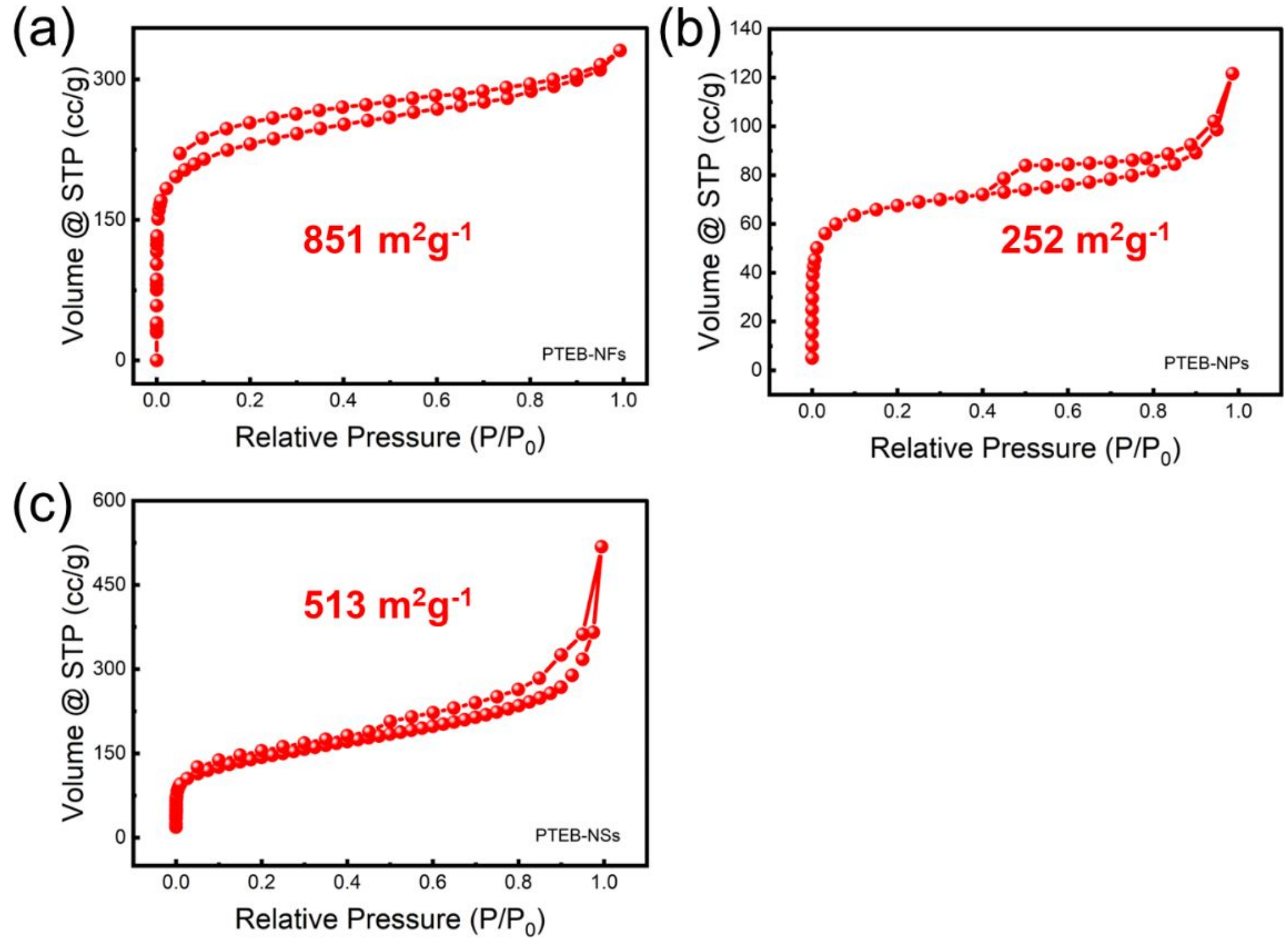

Figure S3. Nitrogen adsorption-desorption isotherm of PTEB-NFs (a), PTEB-NPs (b) and PTEB-NSs (c).

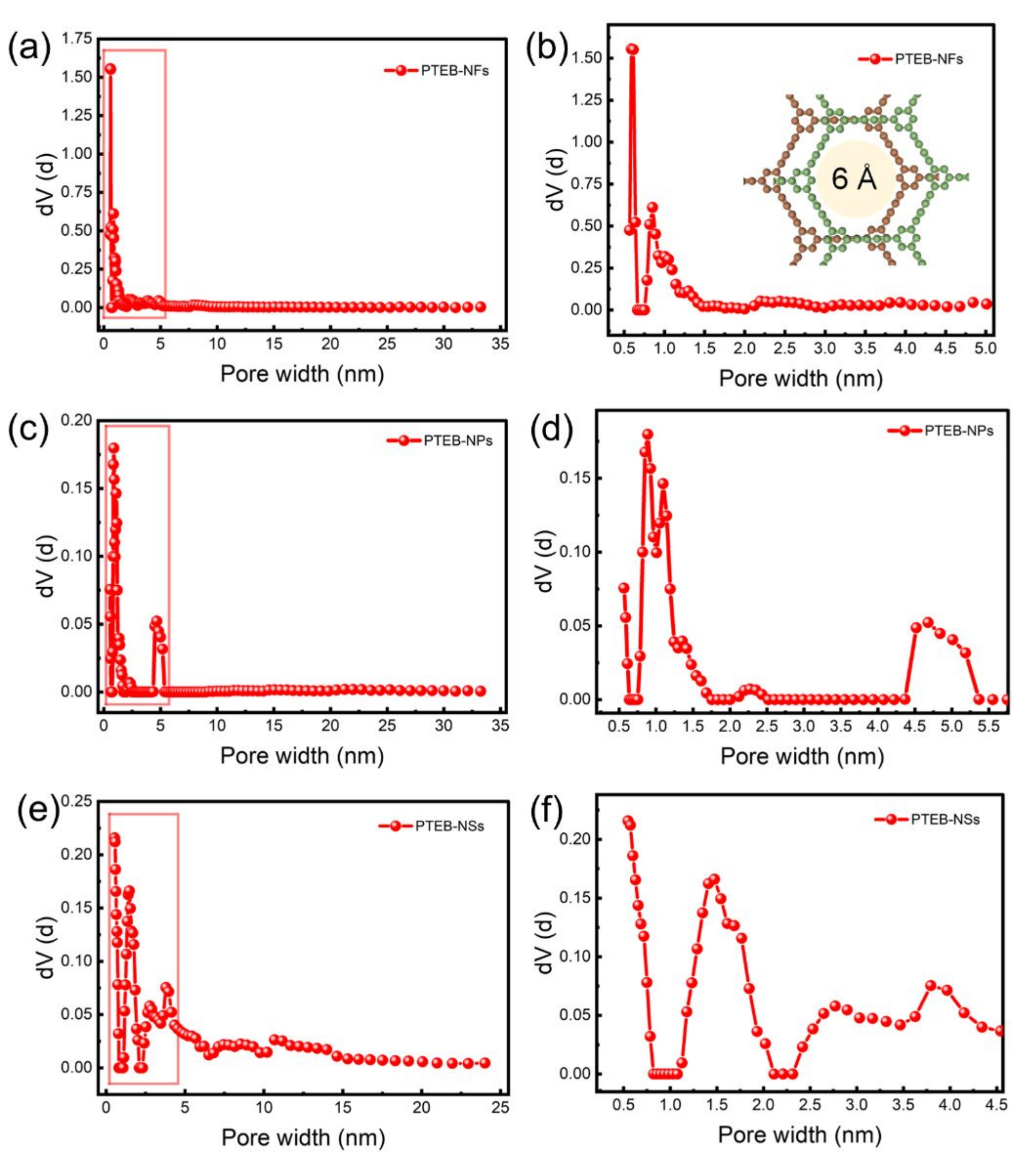

Figure S4. BET pore width distribution of PTEB-NFs (a-b), PTEB-NPs (c-d) and PTEB-NSs (e-f). It is obvious that NF samples have the smallest pore size distribution and the highest specific surface area.

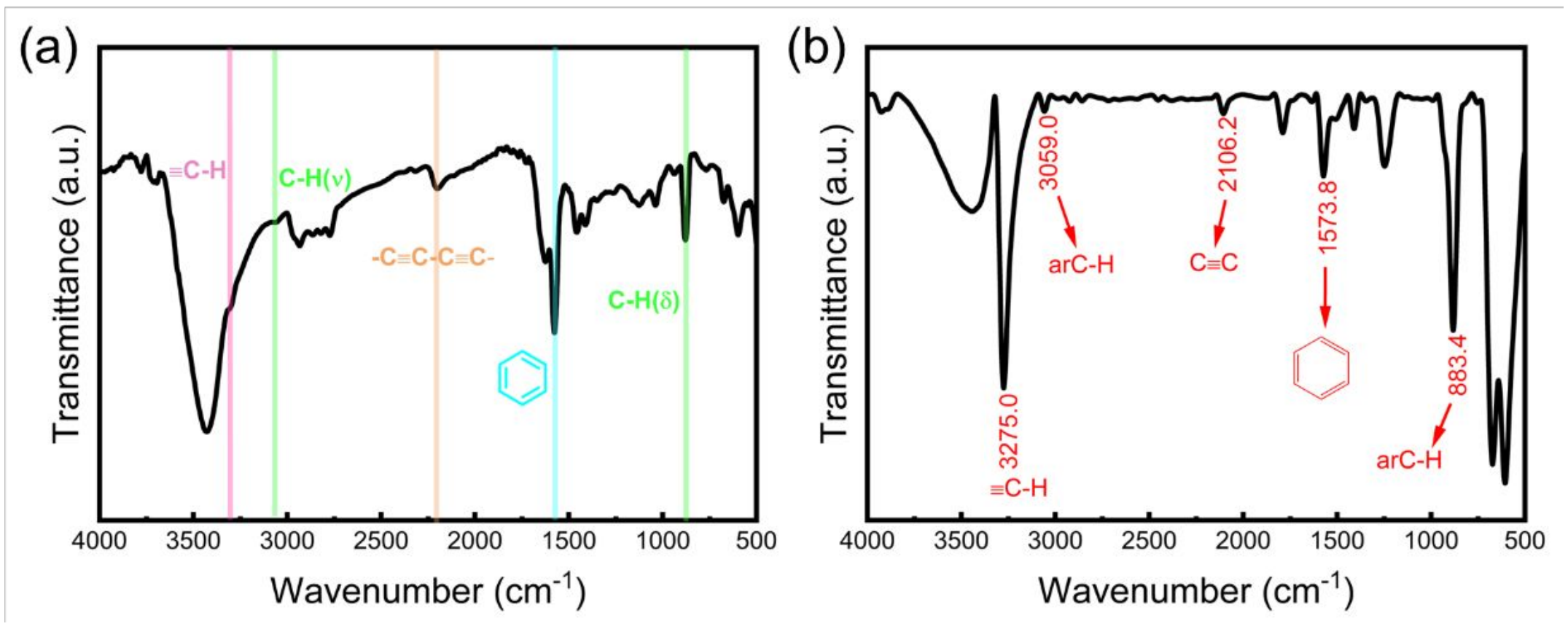

Figure S5. The FTIR spectrum of PTEB-NFs and TEB monomer.

Compared with the TEB spectrum in Figure S5b, the appearance at the wavelength of 2200 cm^{-1} demonstrates the emergence of the butadiyne structure (-C≡C-C≡C-) and also represents the successful coupling of the PTEB polymer.[21-22] Besides, it is also clearly observed the decreasing intensity of the acetylenic C-H vibration at 3275 cm^{-1} which is corresponding to the cross-coupling reaction of TEB.[23]

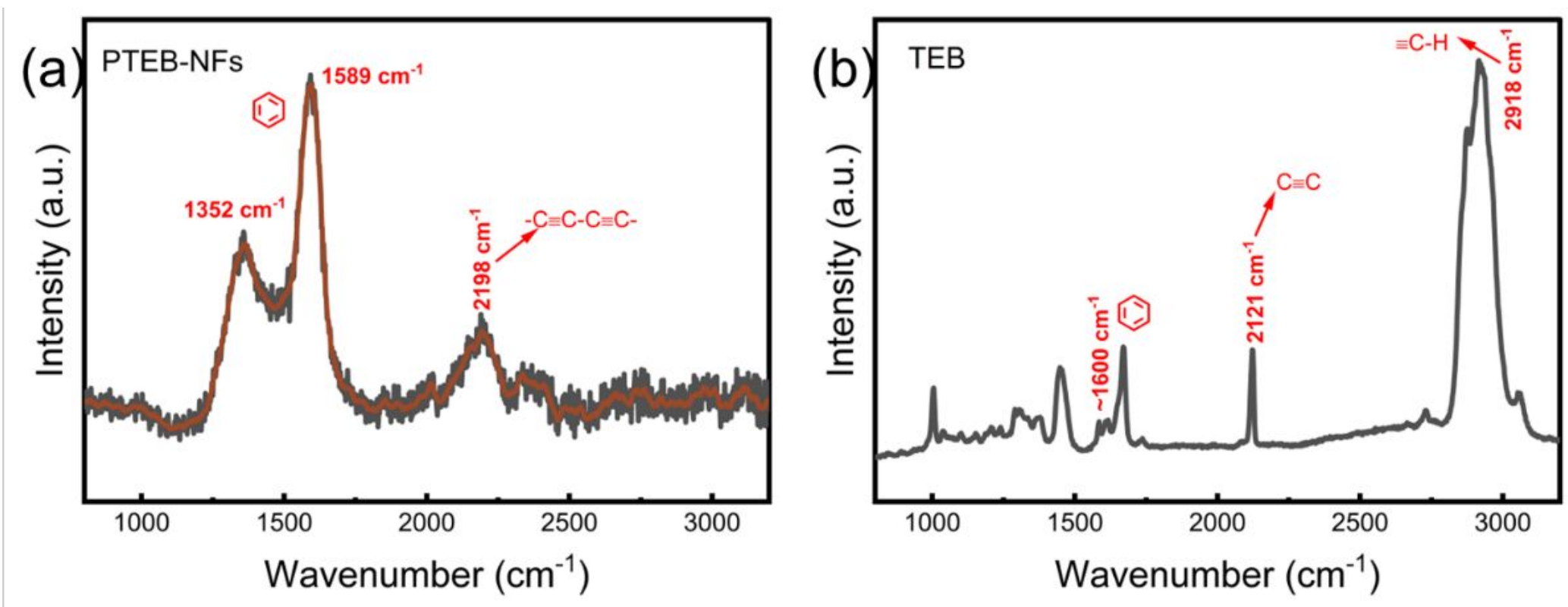

Figure S6. The Raman spectrum of PTEB (a) and TEB monomer (b).

Similar to the FTIR results, the appearance of the 2198 cm^{-1} peak and the absence of the acetylenic C-H bond at the 2918 cm^{-1} position in the Raman spectra, as compared to the spectra of the TEB monomer, demonstrate the formation of the polymer.

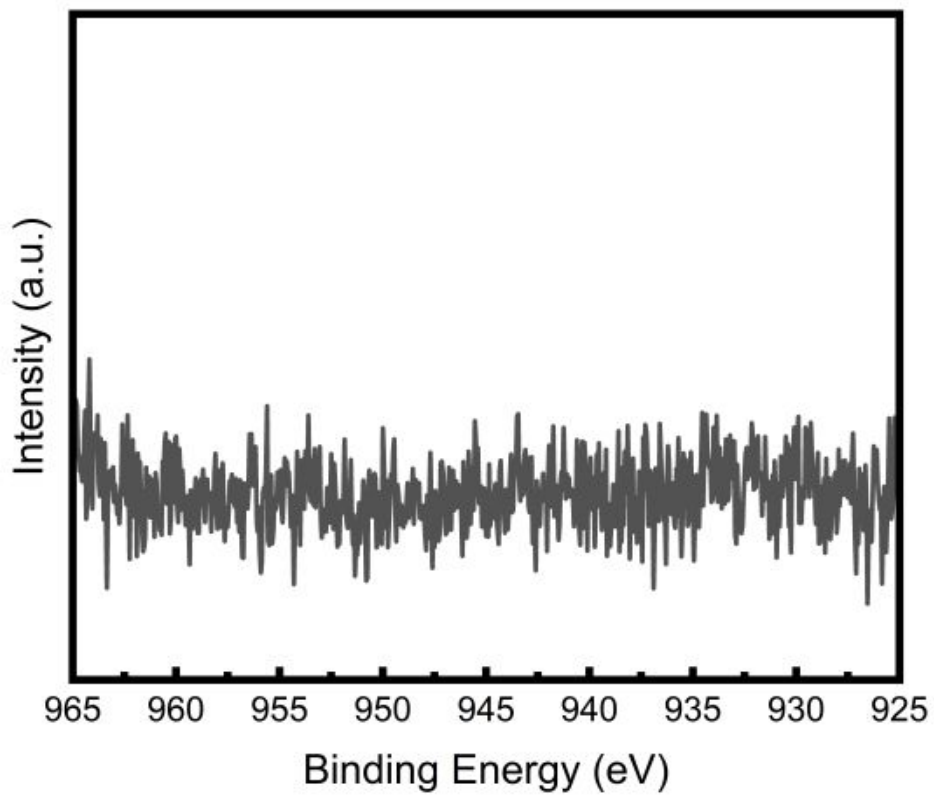

Figure S7. Cu 2p XPS spectrum of Pd/PTEB-NFs, showing no obvious Cu signal and confirming negligible Cu residues.

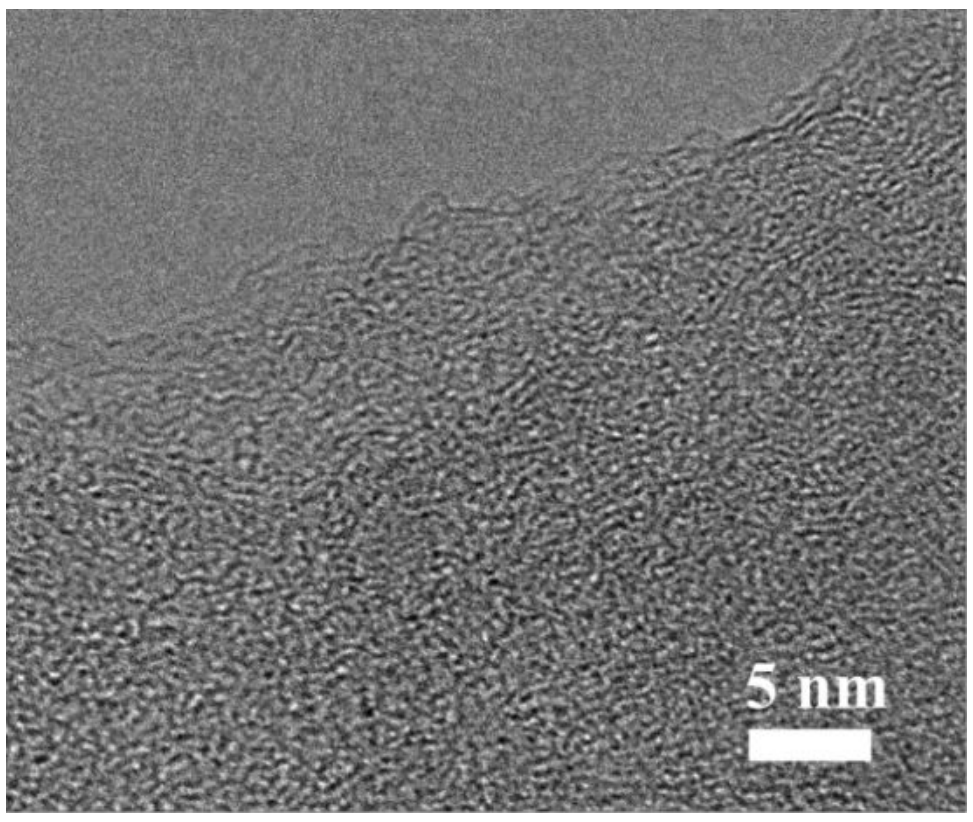

Figure S8. High-resolution Transmission Electron Microscopy (HRTEM) image of (a)PTEB-NFs.

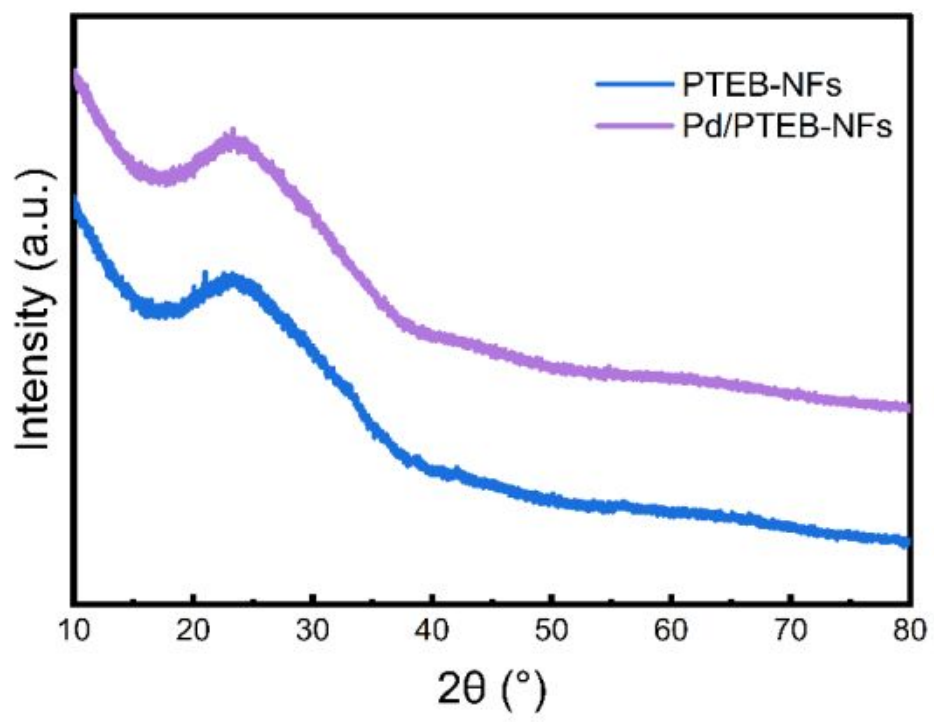

Figure S9. XRD pattern of PTEB-NFs and Pd/PTEB-NFs.

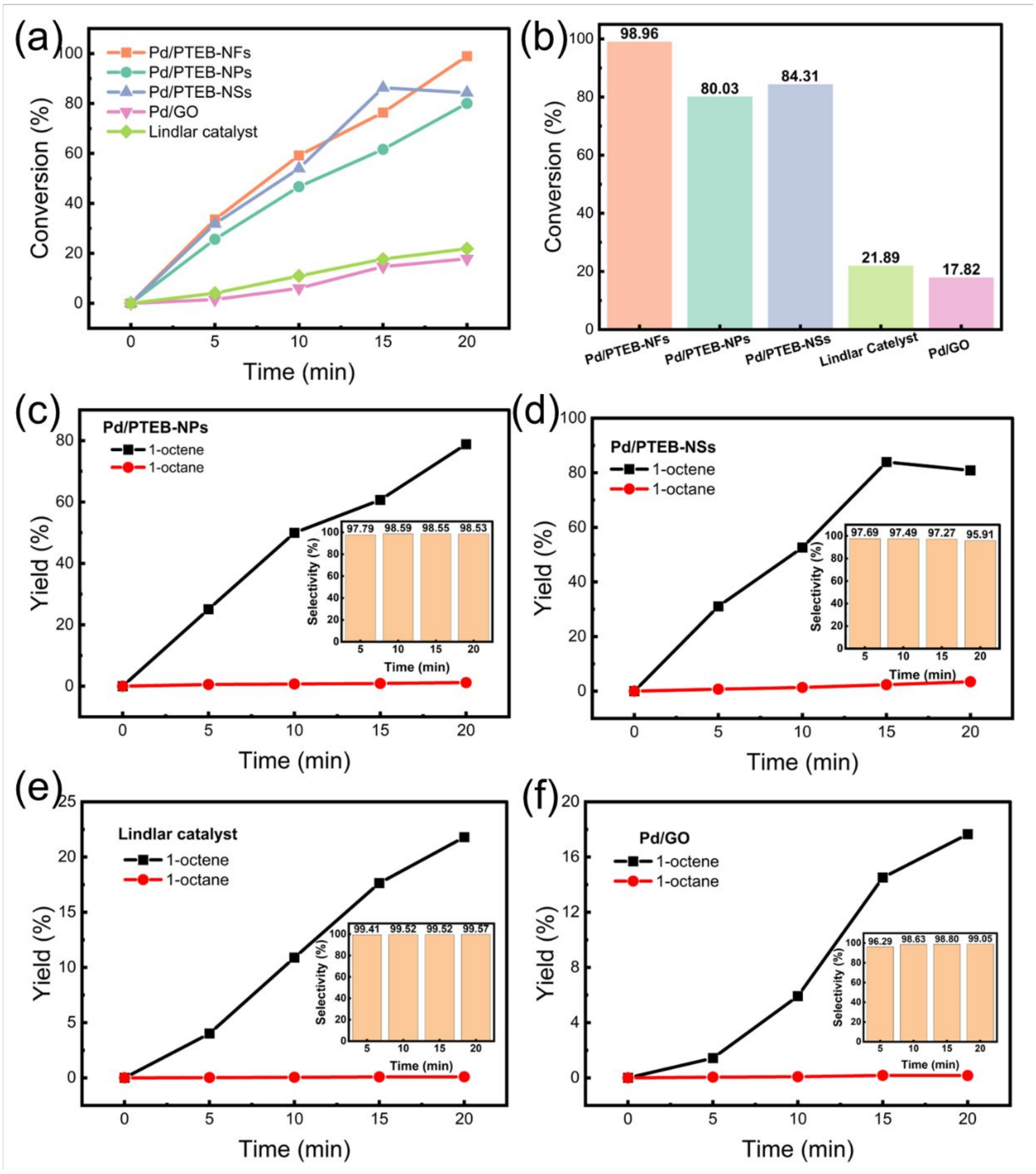

Figure S10. Plots of conversion of 1-octyne versus reaction time by catalysts (a). Conversion of 1-octyne at 20 min reaction time by catalysts (b). Plots of yield of 1-octene and 1-octane versus reaction time catalyzed by PTEB-NPs (c), PTEB-NSs (d), Lindlar catalyst (e) and Pd/GO (f) (Inset: corresponding selectivity versus time).

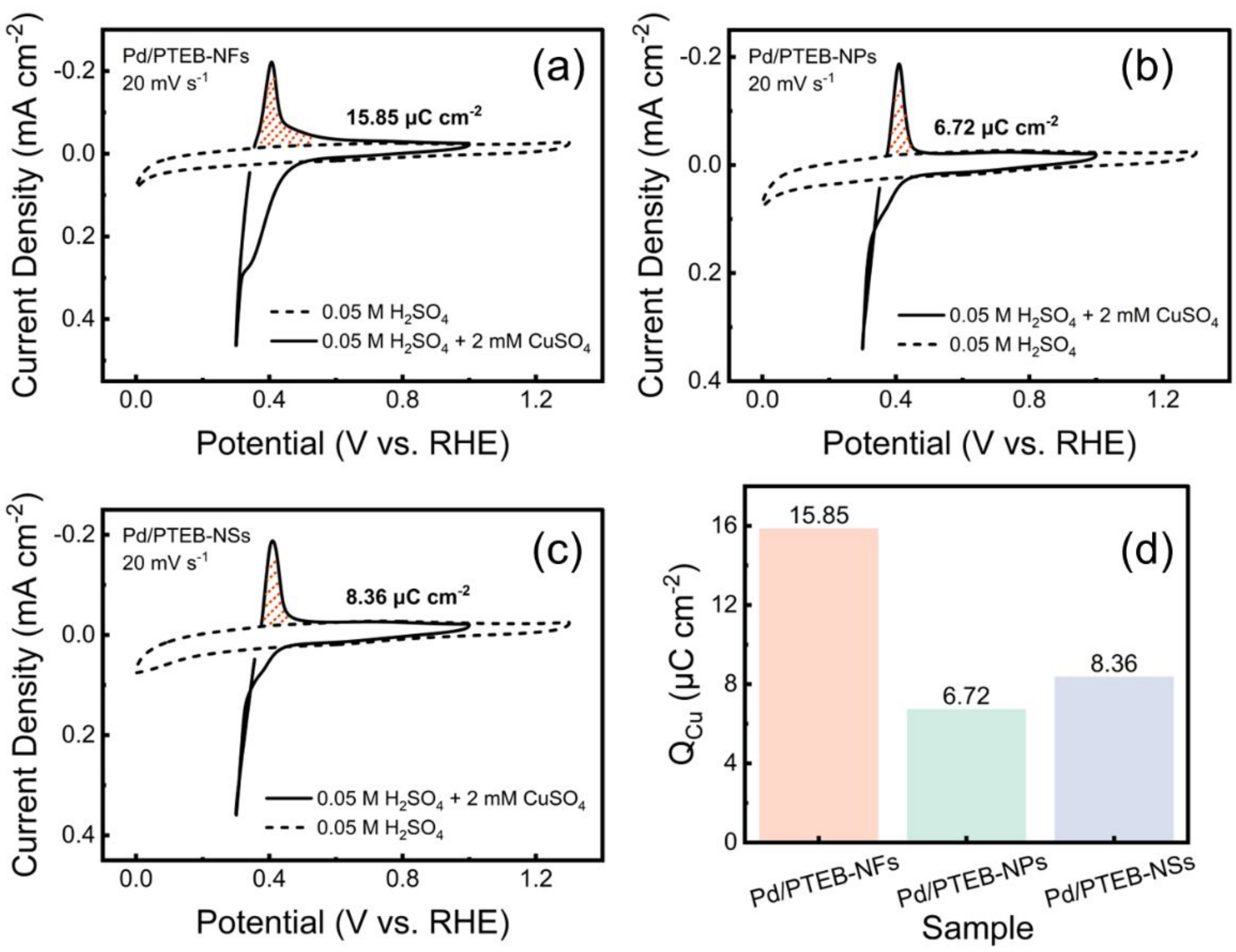

Figure S11. Underpotential deposition of Cu for measuring the surface area of (a) Pd/PTEB-NFs, (b) Pd/PTEB-NPs and (c) Pd/PTEB-NSs. (d) Bar chart comparison of Cu dissolution charge value (Q_{Cu}) of Pd/PTEB samples.

For the Cu stripping experiments, a N_2-saturated solution containing 2 mM $CuSO_4$ and 0.05 M H_2SO_4 was used as the electrolyte. The potential was first held at 0.3 V versus RHE for 100 s to form a Cu_{upd} monolayer, and then CVs were obtained from 0.3 V to 1.0 V at 20 mV s^{-1}. The active surface areas were calculated by subtracting the background CVs that were collected in N_2-saturated 0.05 M H_2SO_4 at 20 mV s^{-1}.[24]

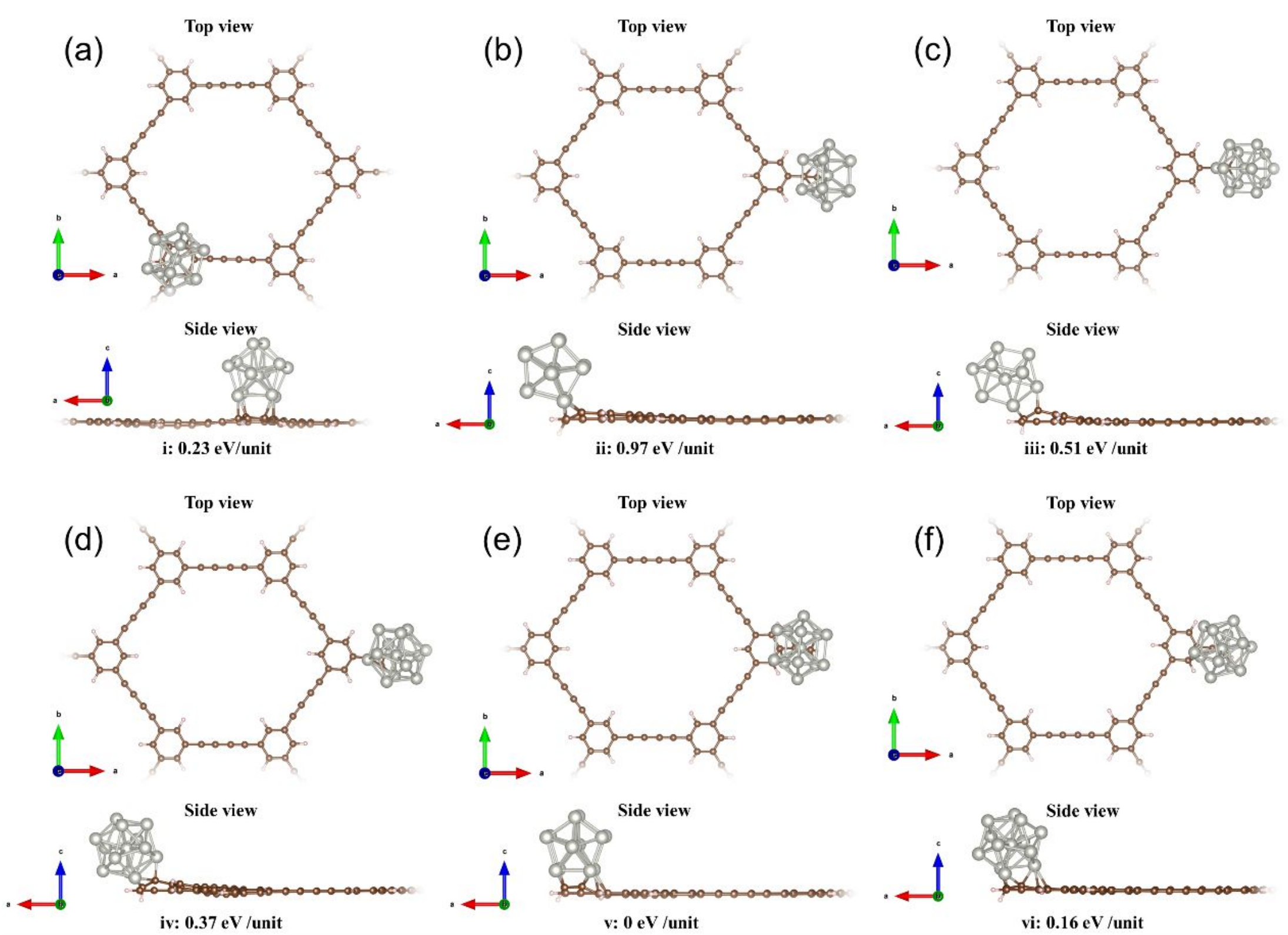

Figure S12. The optimized structural models of PTEB chelated with Pd clusters (i-vi). The grey, brown and pink spheres represent Pd, C, and H atoms, respectively. More in-plane chelating structures like (a) are given in our previous work.[25]

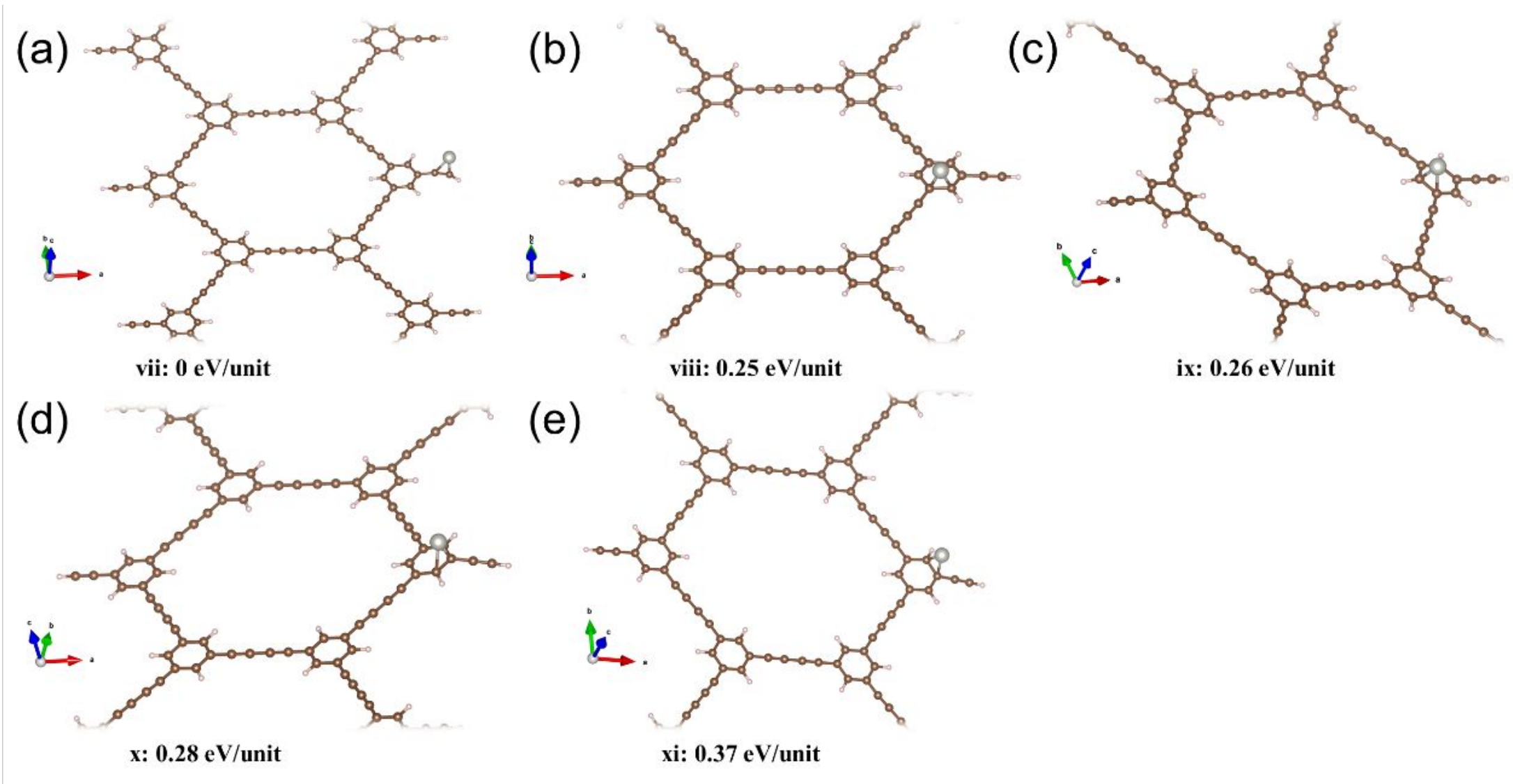

Figure S13. The optimized structural models of PTEB chelated with single atom Pd (vii-xi). The grey, brown and pink spheres represent Pd, C, and H atoms, respectively.

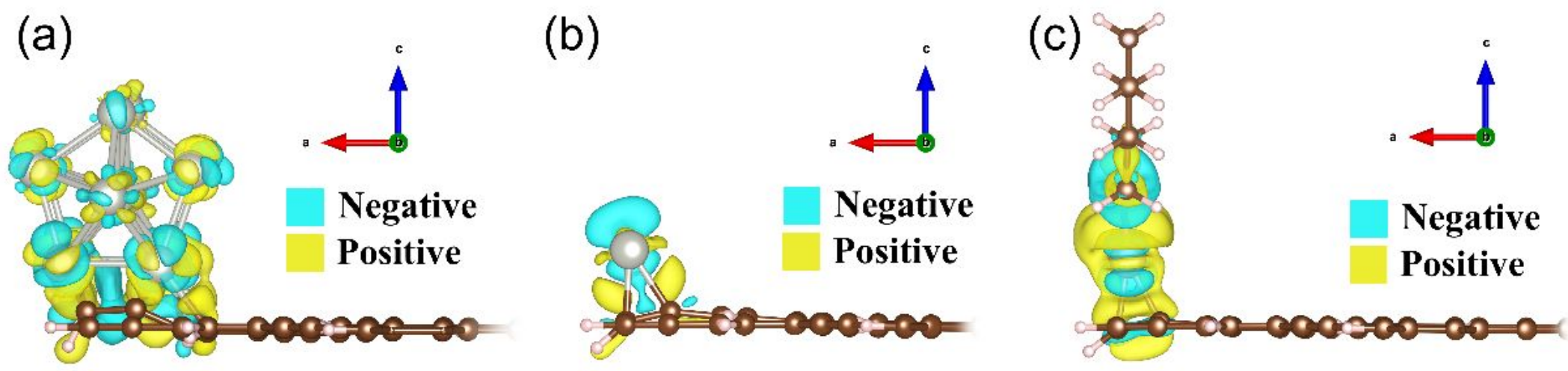

Figure S14. (a) Differential charge density distributions of PTEB anchored with Pd cluster. (b-c) Differential charge density distribution of PTEB anchored with Pd single atom (b) and with 1-octyne adsorbed (c). Yellow and cyan areas indicate electron accumulation and depletion, respectively.

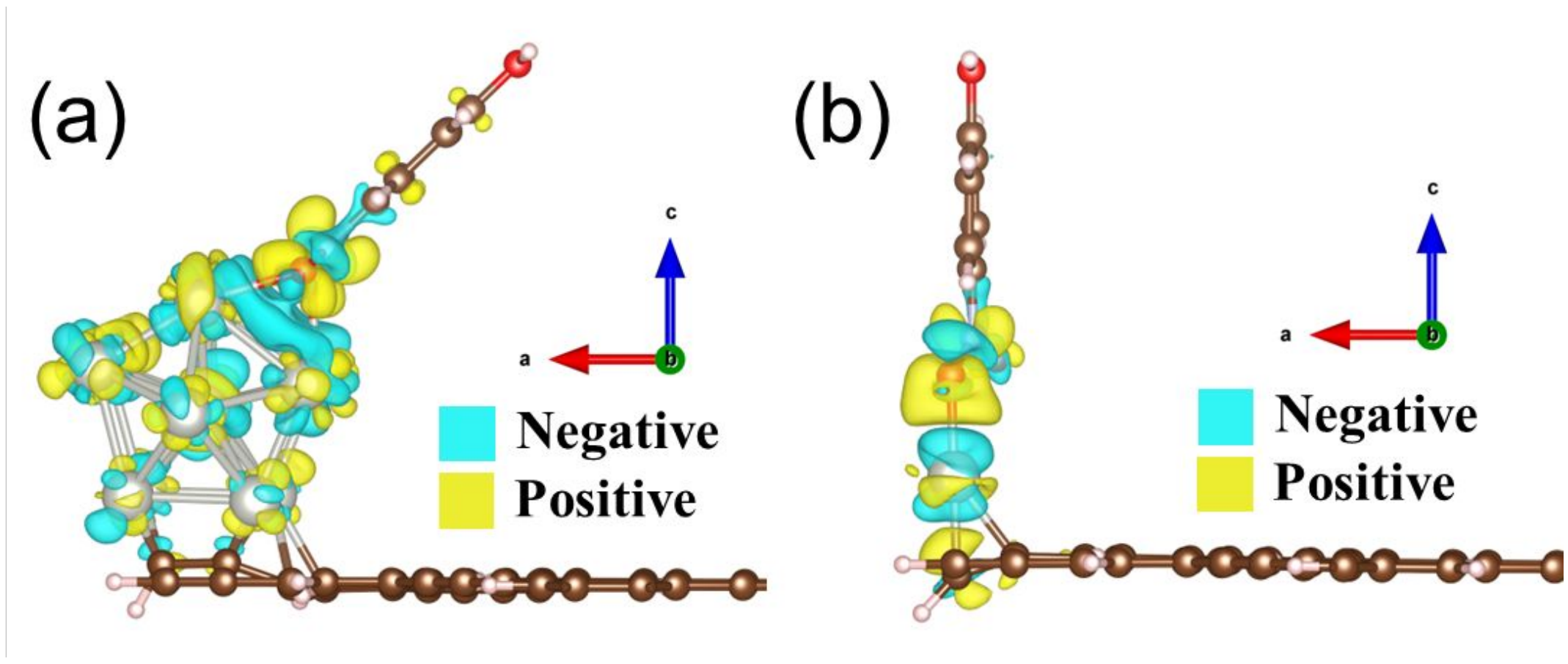

Figure S15. Differential charge density distribution of (a) PTEB anchored by Pd cluster with 4-NP adsorbed and (b) PTEB anchored by Pd single atom with 4-NP adsorbed.

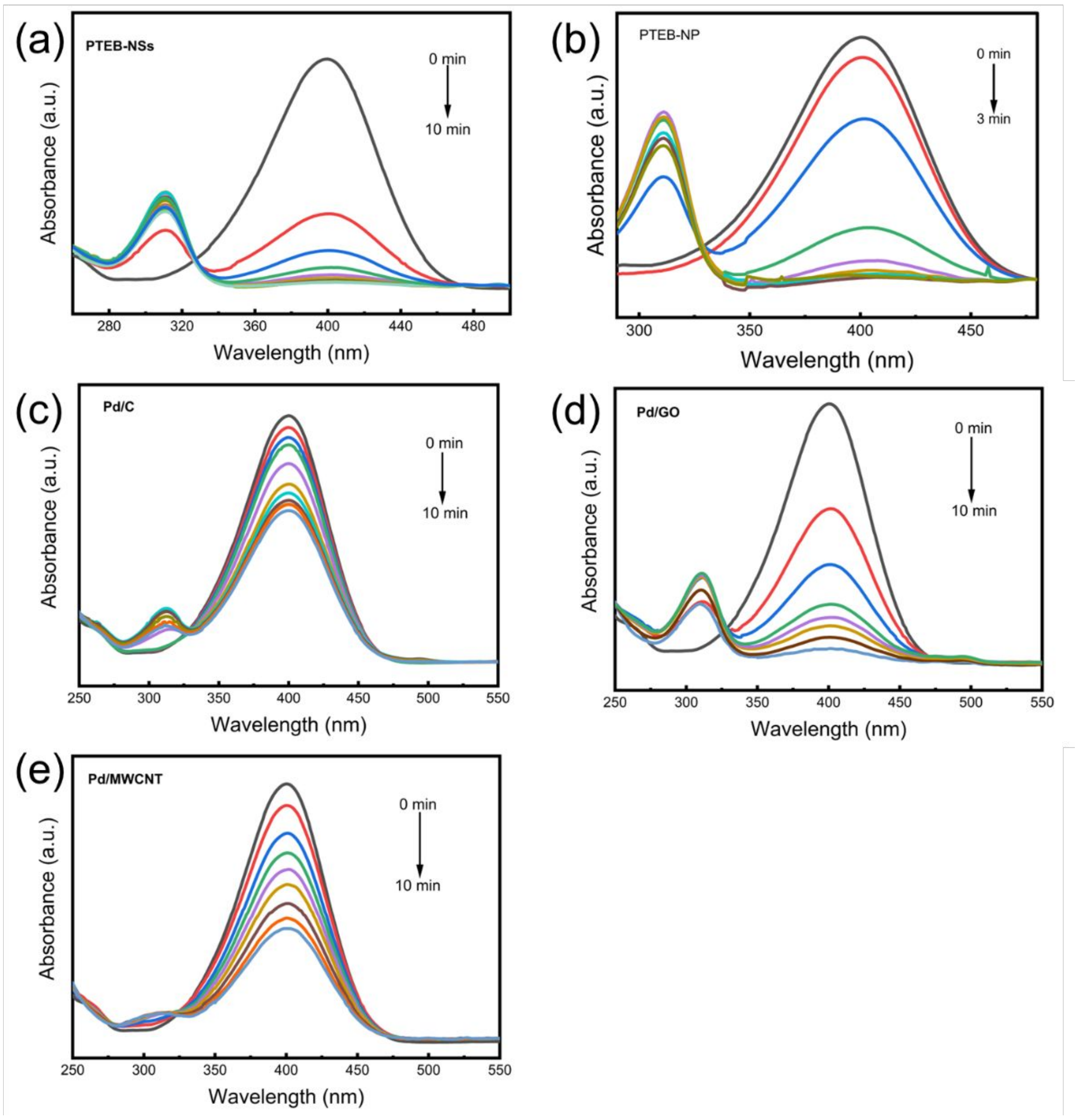

Figure S16. Time-dependent UV-Vis absorption spectra recorded during the 4-NP reduction catalyzed by (a)PTEB-NSs, (b)PTEB-NPs, (c)Pd/C, (d)Pd/GO and (e)Pd/MWCNT. The spectra recorded during the 4-NP reduction catalyzed by PTEB-NFs is displayed in Supplementary Figure 17a.

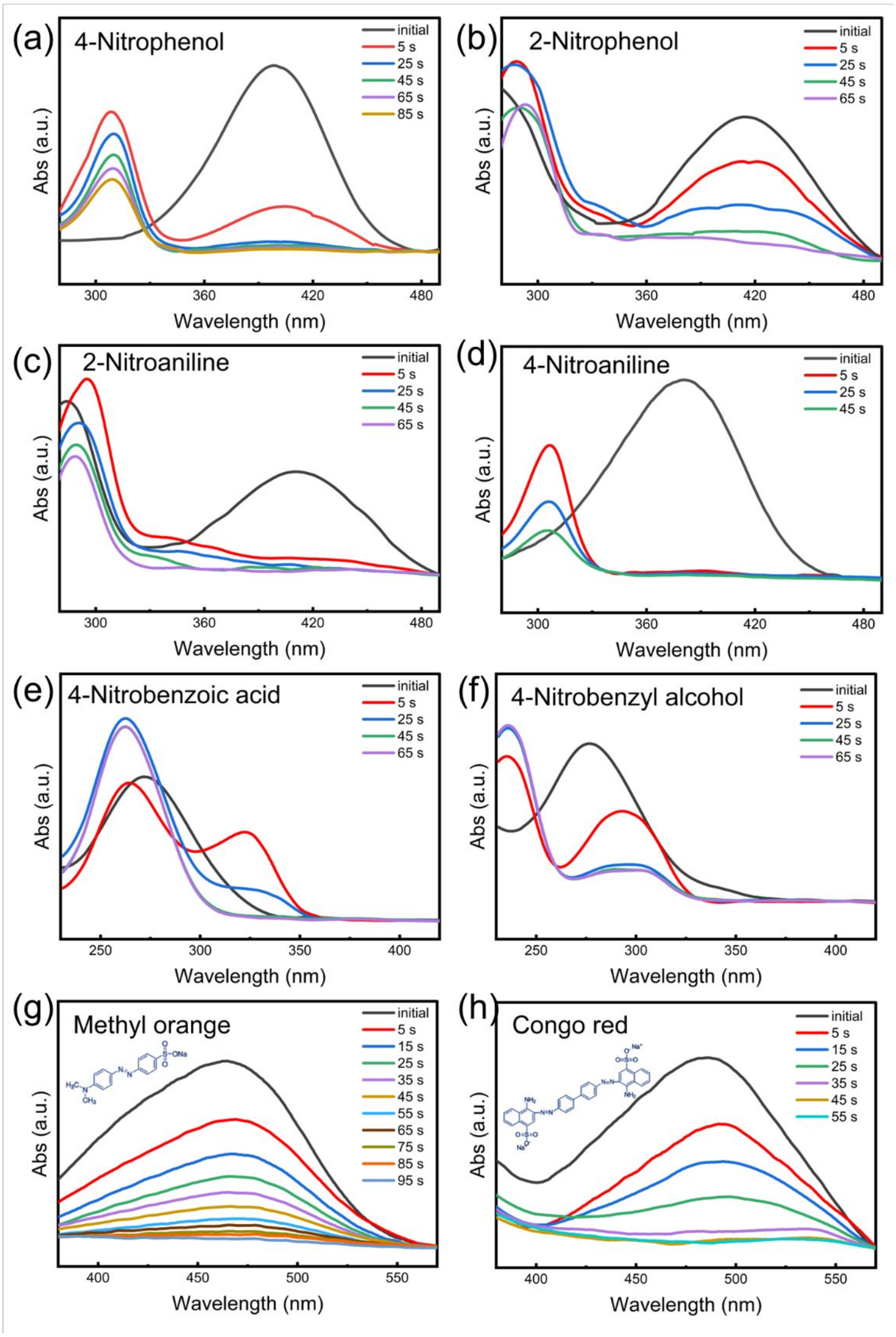

Figure S17. Catalytic hydrogenation of nitroarene derivatives. Time-dependent UV-Vis absorption spectra recorded of (a) 4-nitrophenol, (b) 2-nitrophenol, (c) 2-nitroaniline, (d) 4-nitroaniline, (e) 4-nitrobenzoic acid, (f) 4-nitrobenzyl alcohol, (g) Methyl orange and (h) Congo red.

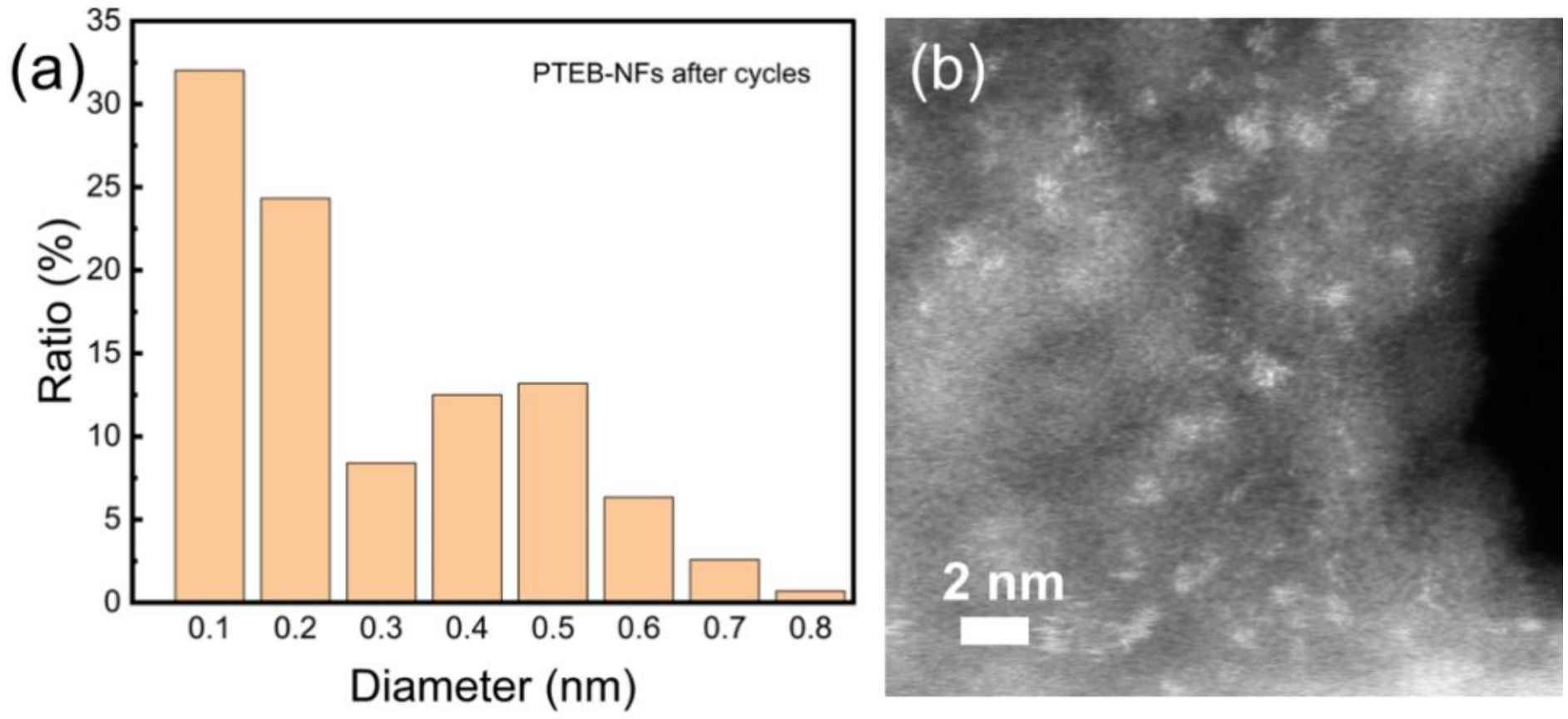

Figure S18. The (a) statistical result of Pd diameters and (b) AC-STEM image of PTEB-NFs after cyclic performance test, suggesting that no significant aggregation occurred after cyclic testing.

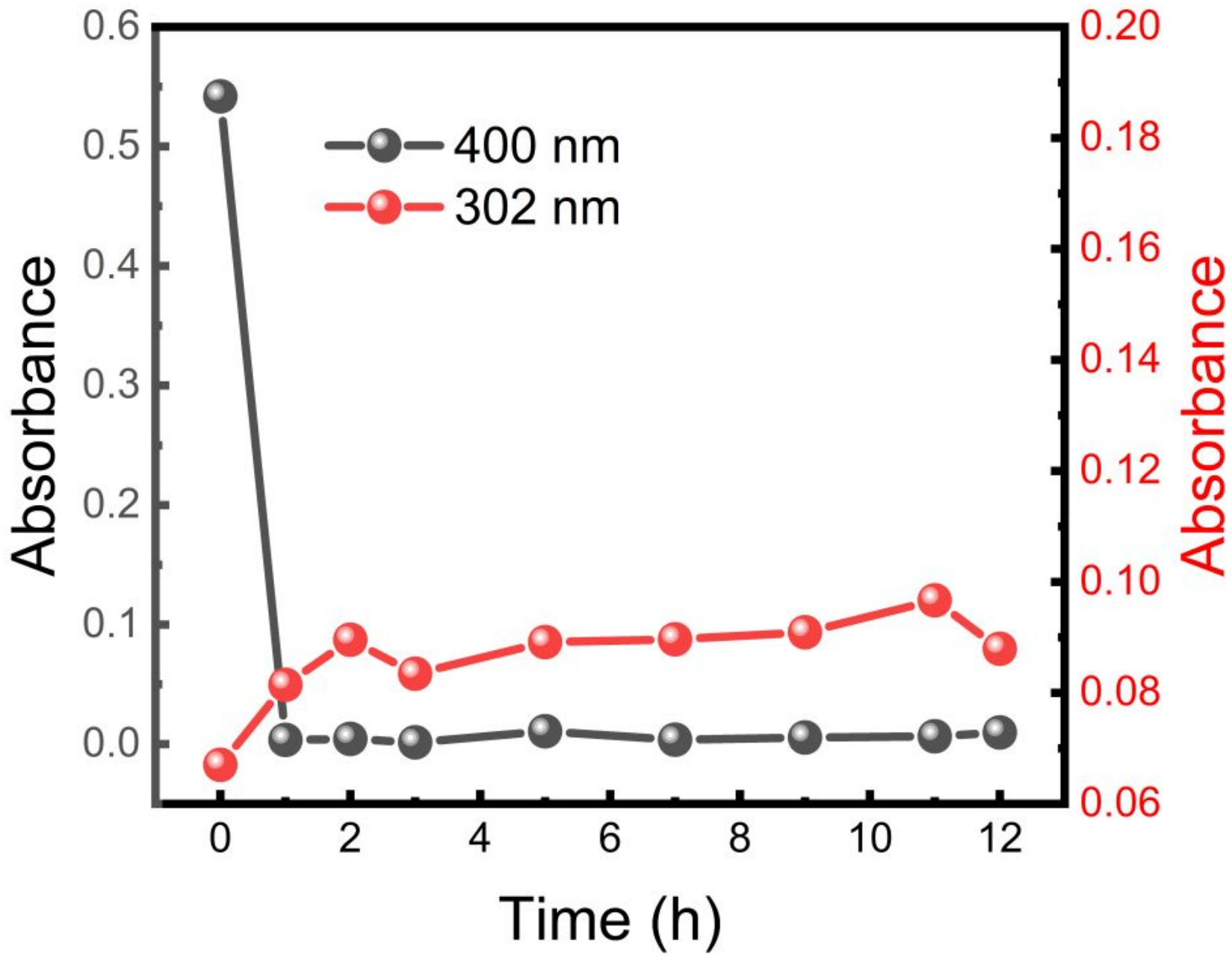

Figure S19. plot curve of characteristic peak intensity of 4-NP and 4-AP versus time

A continuous filter extraction system is constructed to further evaluate the persistent catalytic activity of PTEB-NFs which may be promising for practical applications (Figure 4d). The 4-NP/$NaBH_4$ solution turns into colorless after passing through the catalyst film at ca. 8 mL min^{-1} flow rate (Supplementary video). According to Figure S19, no catalytic activity degradation has been found since the absorbance of 4-NP at 400 nm does not increase over a 12 h period, indicating exceptional endurance of PTEB-NFs during the reduction process.

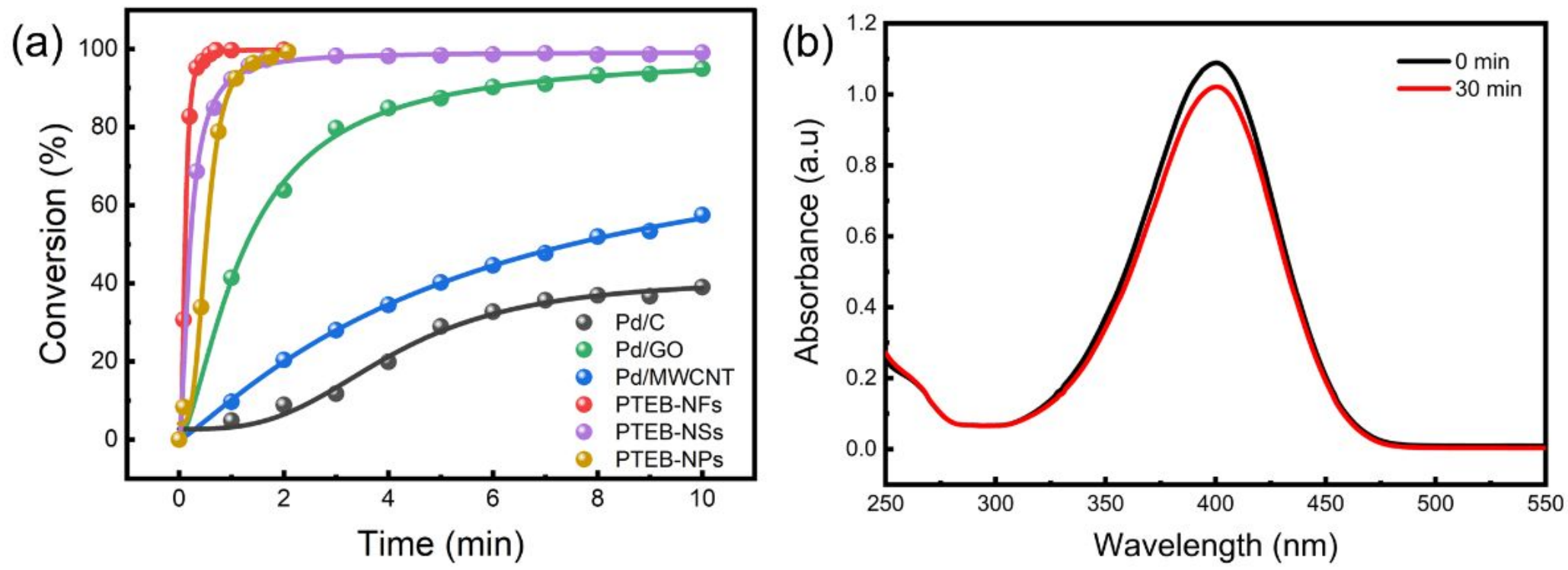

Figure S20. Comparative catalytic conversion as a function of time for the reduction of 4-NP catalyzed by different catalysts (a); Time-dependent UV-Vis absorption spectra recorded during the 4-NP reduction without catalyst adding (b).

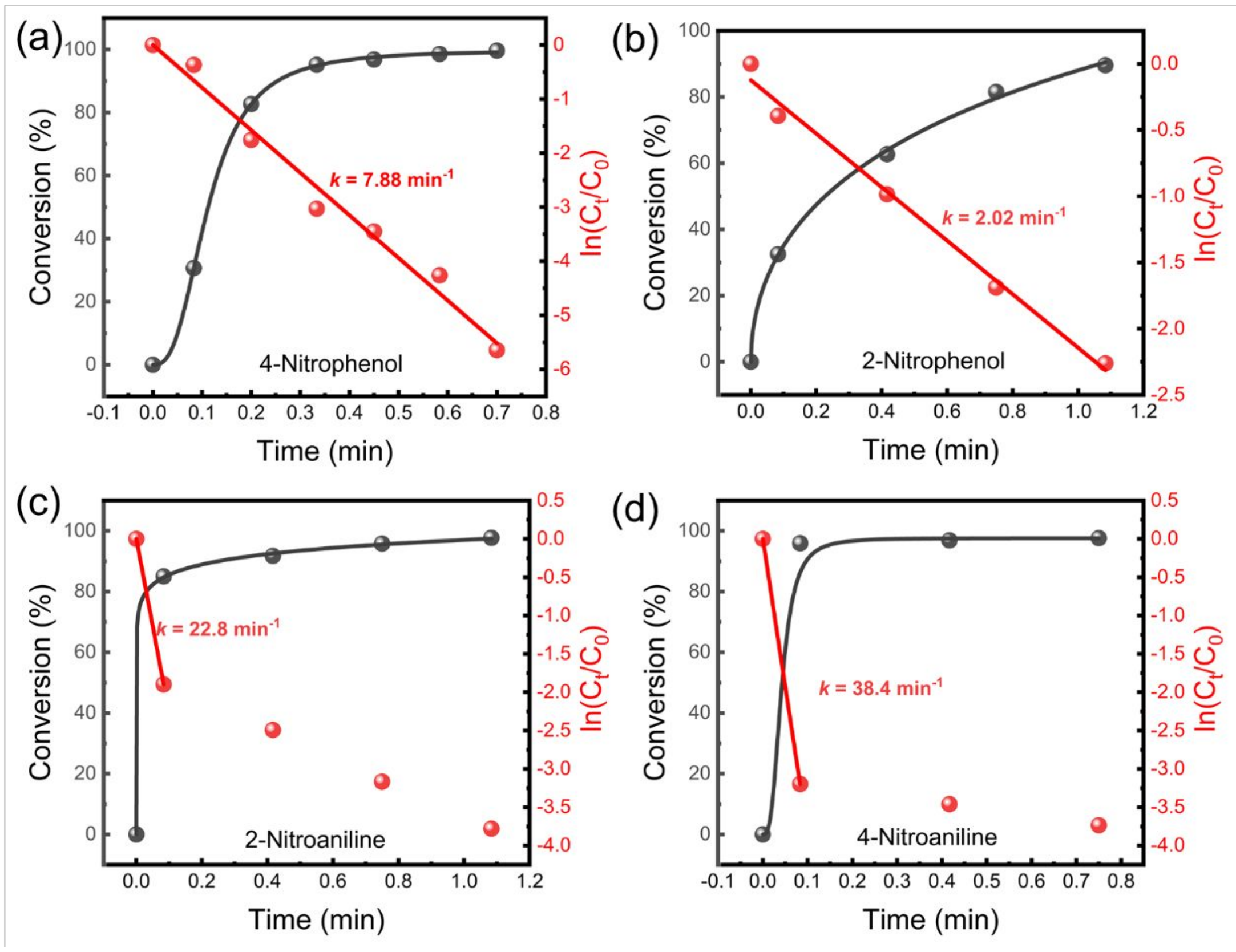

Figure S21. The catalytic conversion and plots of $\ln(C_t/C_0)$ of absorbance of (a) 4-nitrophenol, (b) 2-nitrophenol, (c) 2-nitroaniline and (d) 4-nitroaniline at their corresponding characteristic peak obtained from Supplementary Figure 17.

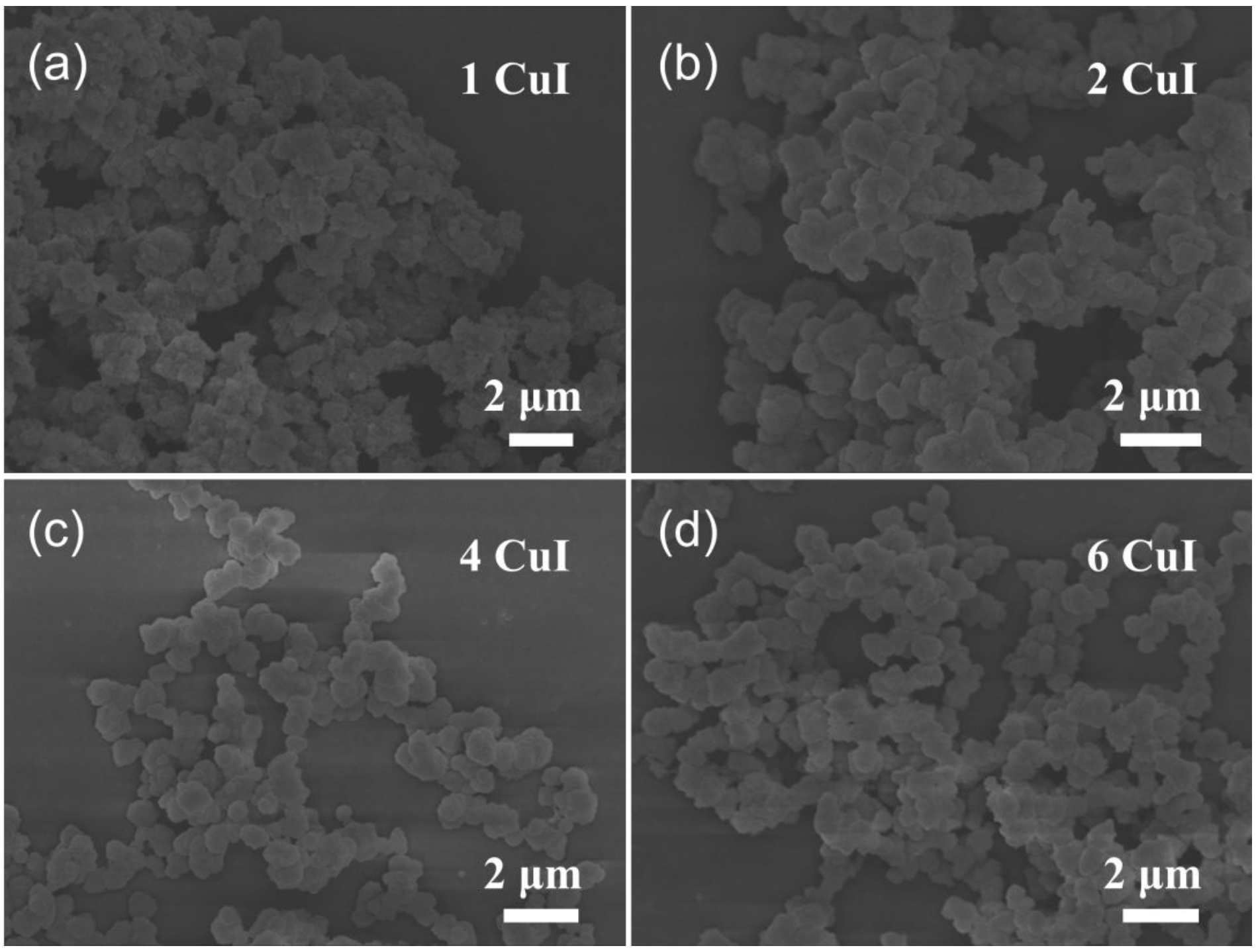

Figure S22. SEM images of corresponding product obtained by adding different amounts of CuI under the same conditions 48 h (O_2 as the oxidant).

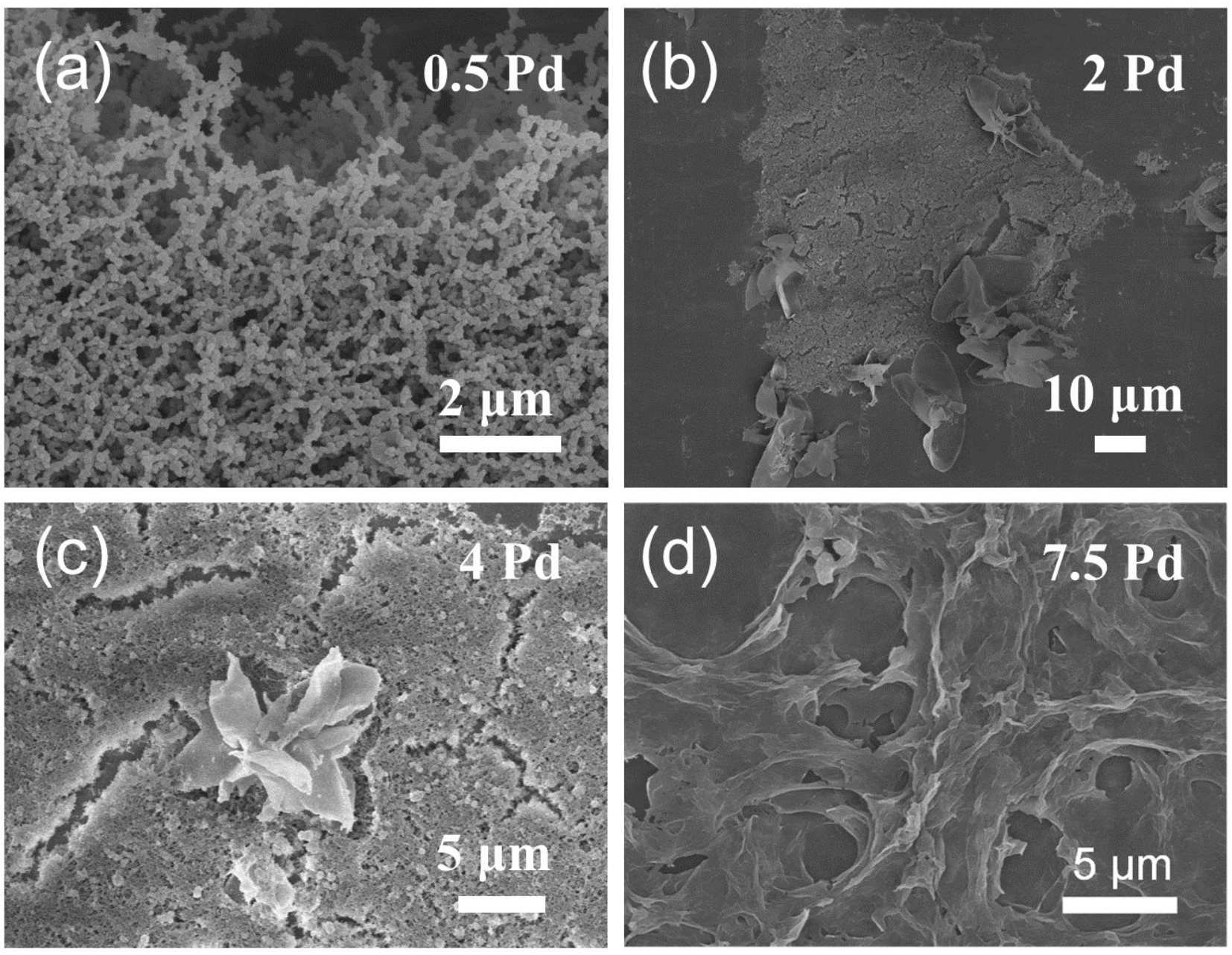

Figure S23. SEM images of corresponding product obtained by adding different amounts of $PdCl_2(PPh_3)_2$in an argon atmosphere 48 h (*p*-benzoquinone (PBQ) as the oxidant).

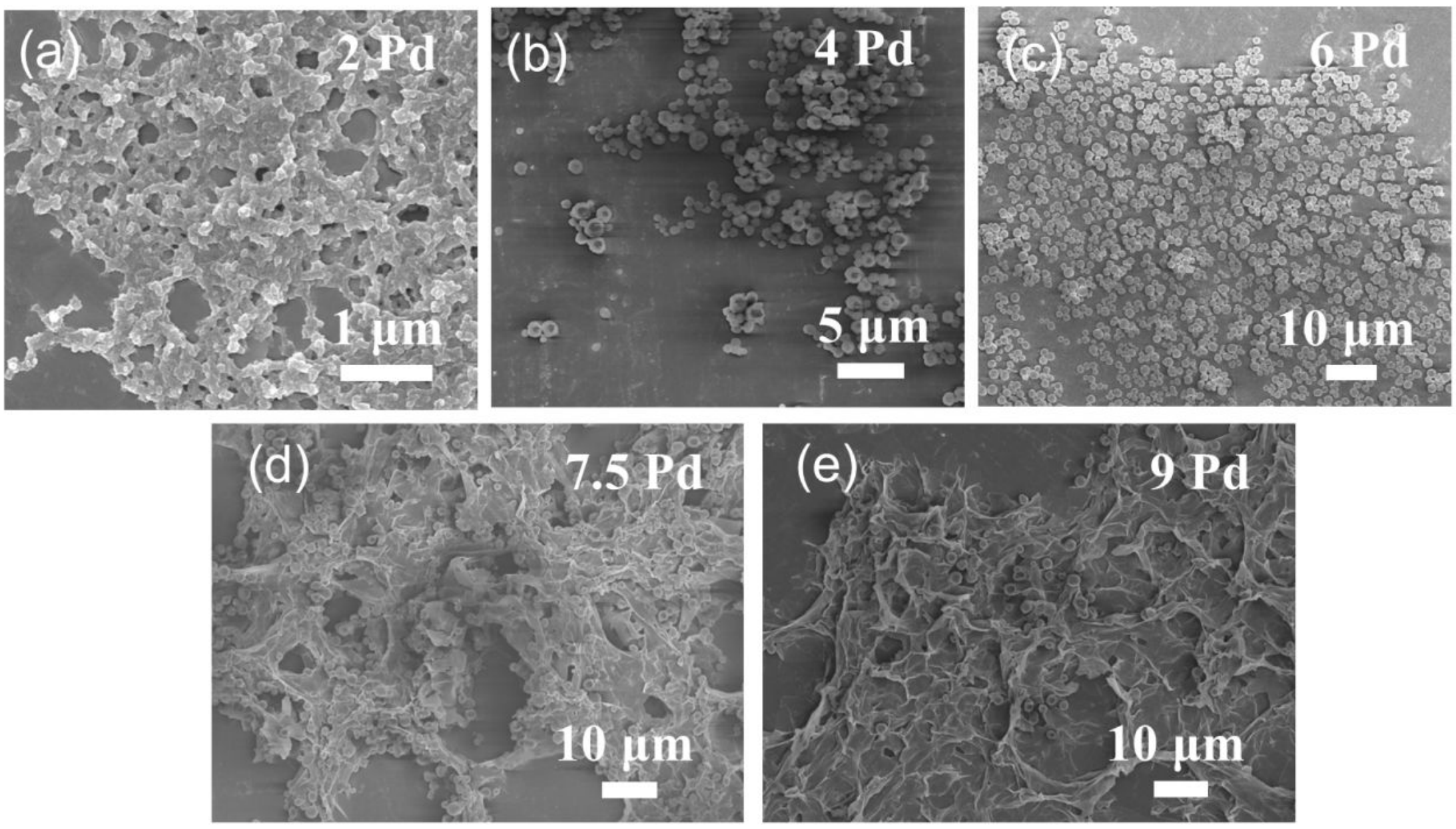

Figure S24. SEM images of corresponding product obtained by adding different amounts of $PdCl_2(PPh_3)_2$in an argon atmosphere 48 h (I_2 as the oxidant). Similar morphological evolution in I_2 oxidant system require higher Pd catalyst concentrations to initiate, which is ascribed to the numerous side reactions with terminal acetylenes resulting inhomogeneous polymers of low molecular weights and lower conversion Pd(0) to Pd(II).[15-16]

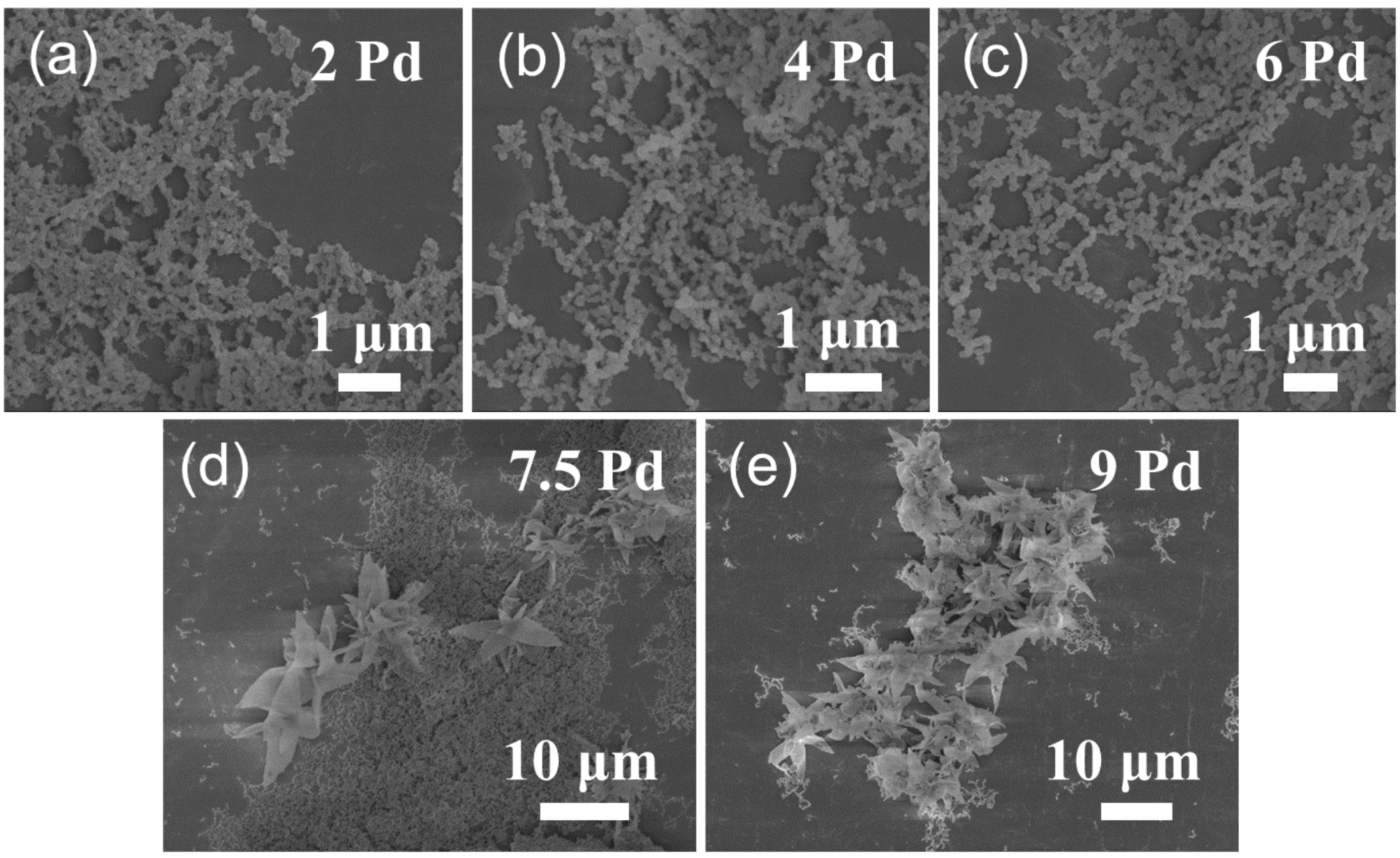

Figure S25. SEM images of corresponding product obtained by adding different amounts of $PdCl_2(PPh_3)_2$ in an argon atmosphere 48 h (p-naphthoquinone (PNQ) as the oxidant).

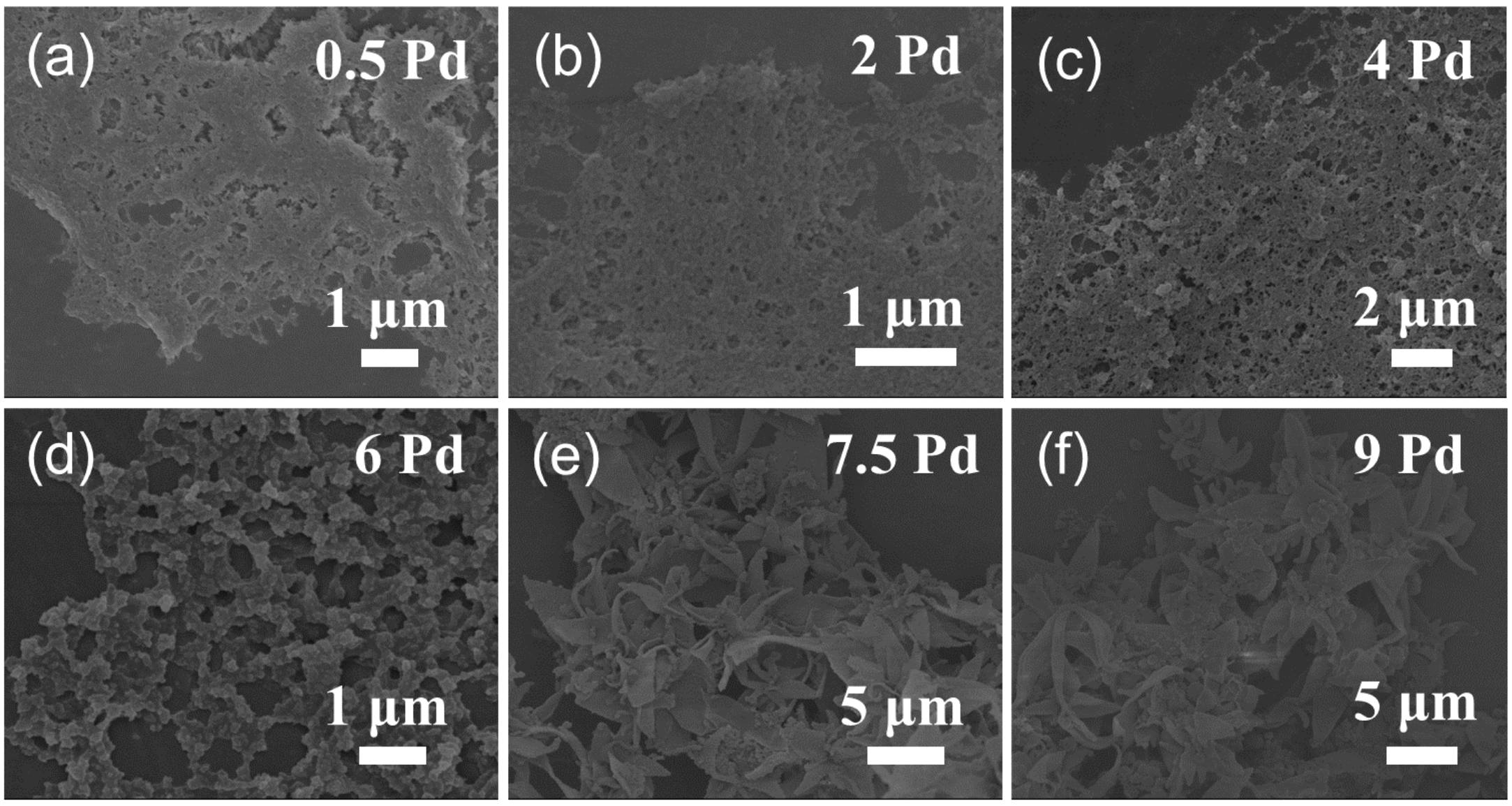

Figure S26. SEM images of corresponding product obtained by adding different amounts of $PdCl_2(PPh_3)_2$ in an argon atmosphere 48 h (anthraquinone (AQ) as the oxidant).

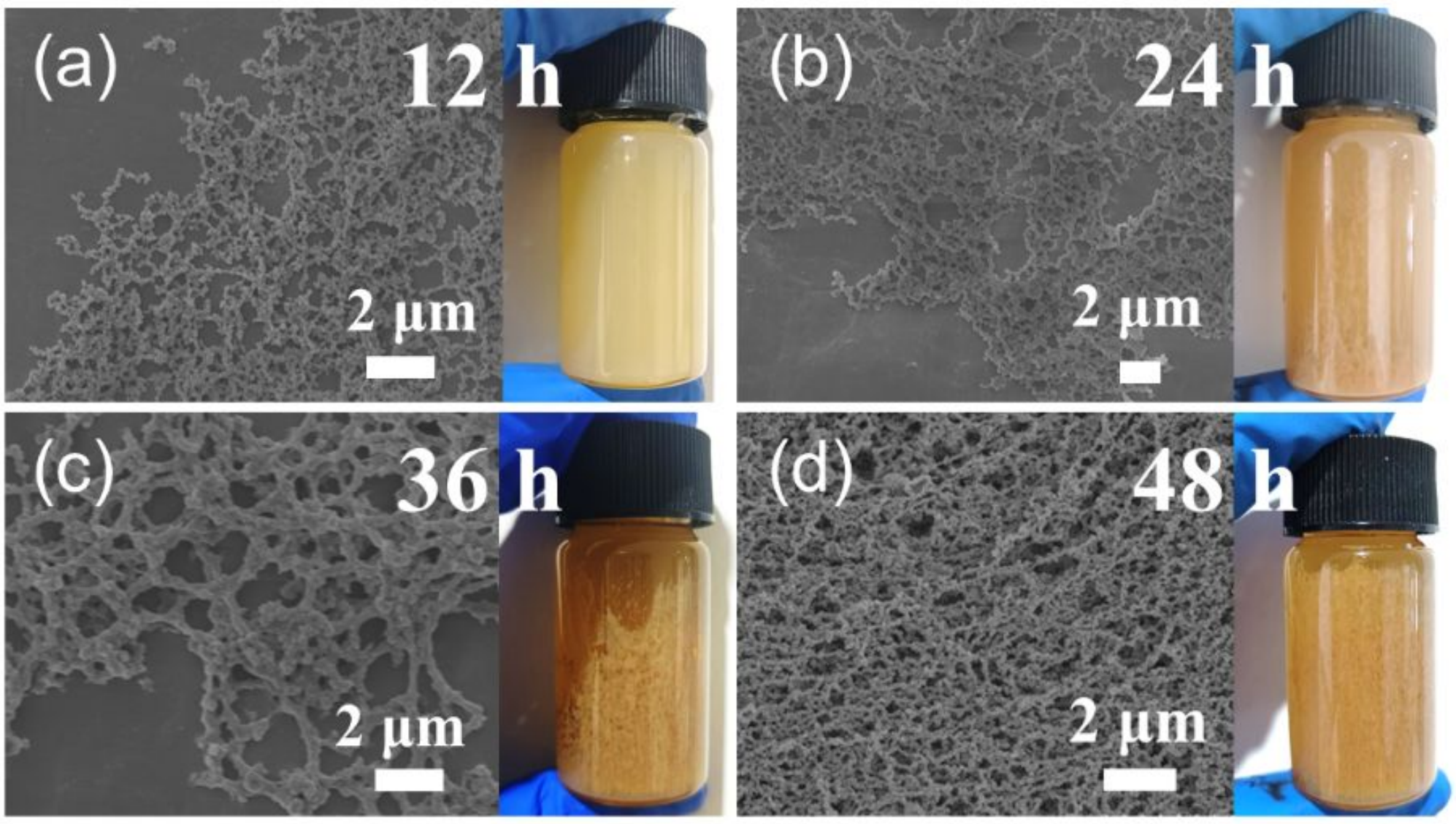

Figure S27. SEM images of PTEB-NFs samples over time and corresponding photos. It can be seen that with the extension of reaction time, the generated PTEB-NFs structure is gradually

extended and stacked by a small number of coupled short chains, and finally self-assembled into an interconnected network with a three-dimensional network structure. The macroscopic picture also gradually formed a cross structure with a certain network morphology from the turbidness and shapeless sample solution, which is consistent with the SEM images shows.

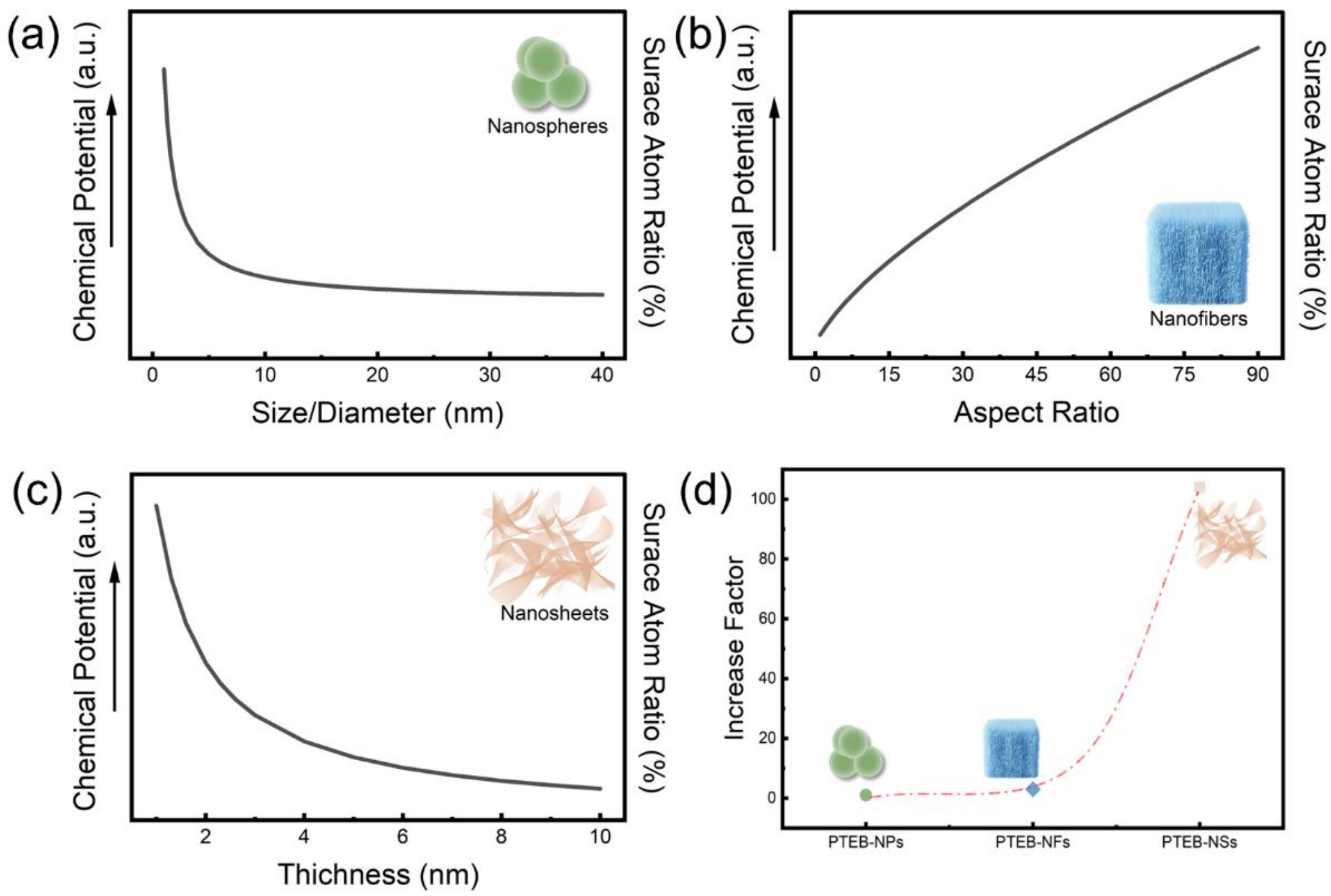

Figure S28. Size dependence of the surface atom ratio and the relative chemical potential of difference nanostructures. (a) assuming a spherical shape. (b) Shape dependent chemical potential of nanofibers and (c) nanosheets. (d) When the nanostructure is elongated from a dot to a rod and a thin rectangle with its surface atom ratio increase by a factor of ca. 3 and 104 respectively. The volume of all nanostructures is set to the same value. The thickness of nanosheet is set at 2 nm according to the previous literature reported by us.[25]

Definition of conversion, yield, and selectivity in catalytic reactions

$$\text{Conversion} = \frac{\text{Moles of reactant consumed}}{\text{Moles of reactant fed in system}} \times 100\% \tag{S8}$$

$$\text{Yield} = \frac{\text{Actual moles of specific product}}{\text{The maximum moles of specific product (fully produced)}} \times 100\% \tag{S9}$$

$$\text{Selectivity} = \frac{\text{Moles of desired product}}{\text{Moles of reactant consumed}} \times 100\% \tag{S10}$$

Table S1. Summary of the relative amounts of Pd chelated in series of samples according ICP-OES results.

Sample	Pd catalyst loadings	Morphology	Relative amounts of Pd (wt%)
1	0.5 mg	NFs	0.70
2	2 mg	NFs	3.17
3	4 mg	NFs	6.34
4	7.5 mg	NSs	7.80
5	0.5 mg	NPs	1.02

Table S2. Pd K-edge EXAFS fitting results of synthesized PTEB-NFs catalysts.

**Sample**	**Shell**	**R (Å)[a]**	**C.N.[b]**	**ΔE_0 (eV)[c]**	**σ^2 (*10^{-3} $Å^2$)[d]**	**S_0^2[e]**	**R factor**
Pd foil	Pd-Pd	2.74±0.01	12	-6.0±0.4	5.4	0.78±0.04	0.003
PTEB-NFs (Pd)	Pd-C	1.93±0.01	1.4±0.5	-1.3±0.6	6.8	0.78	0.0083
	Pd-Pd	2.75±0.02	6.1±0.5		7.0		

Note: a. Bond length; b. Average coordination number; c. Difference of potential of sample and standard; d. Debye-Waller factor; e. Amplitude reduction factor;

Table S3. Comparison of catalytic performance of metal-catalyzed semi-hydrogenation of 1-octyne

Catalyst	Reaction conditions	Metal and substrate amount	Catalytic performance	TOF value ($mol_{C=C}$ mol_{metal}^{-1} h^{-1})	Reference
Pd/PTEB-NFs	1 atm, 75 ℃, ethanol	0.39 μmol 1.5 mmol	99% conversion within 20 min; Selectivity: 98.5%.	15039	This work
Pd/PTEB-NPs	1 atm, 75 ℃, ethanol	0.50 μmol 1.5 mmol	80% conversion within 20 min; Selectivity: 98.5%.	9026	This work
Pd/PTEB-NSs	1 atm, 75 ℃, ethanol	0.66 μmol 1.5 mmol	84% conversion within 20 min; Selectivity: 97.5%.	8475	This work
Pd@Ru NSs	1 atm, RT, ethanol	0.1 μmol 0.5 mmol	100% conversion within 40 min; Selectivity: 79.2%.	5940	[26]
5.6 wt% Pd/Activated charcoal	1.5 bar, RT, n-heptane	5.26 μmol 3.31 mmol	1-Octene selectivity: 21% at a conversion of 97%	~1538	[27]
6.5 wt% Pd/Carbon nanofibers	1.5 bar, RT, n-heptane	6.11 μmol 3.31 mmol	1-Octene selectivity: 68.5% at a conversion of 97%	~1800	[27]
5Ag/Al_2O_3	10 bar, 80 ℃, Hexane	5 wt% 2 wt%	20% conversion within 150 min; Selectivity: 95%.	~564	[28]
1Pd/Al_2O_3	1 atm, 100 ℃, hexane	1.06 wt% 2 wt%	100% conversion at 6 h; Selectivity: 5%.	~4	[29]
c-Ni_4Au/Al_2O_3	20 Pa, 110 ℃, n-heptane	0.9 wt% 2 wt%	Selectivity: 95% at a conversion of 75%	~2331	[30]
c-Ni_3Au/Al_2O_3	20 Pa, 110 ℃, n-heptane	2.3 wt% 2 wt%	Selectivity: 95% at a conversion of 38%	~462	[30]
Pd/Ag	1.0Mpa, 80 ℃,	0.49 μmol 1.69 mmol	100% conversion within 60 min; Selectivity: 52%.	3470	[31]

Abbreviations: NSs: nanosheets; NPs: nanoparticles; NCs: nanocrystals; RT: room temperature; THF: tetrahydrofuran; atm: atmosphere. 1 atm = 1.01 bar = 1.01 × 10^5 Pa.

Table S4. Comparison of catalytic activities of Pd metal-catalyzed reduction of 4-nitrophenol at room temperature.

Catalyst	Metal amount	Substrate amount	$\frac{n(4-NP)}{n(Pd)}$	Rate constant (k)	Rate constant per unit mass (k`)	Reference
PTEB-NFs	0.35 μg	0.1 μmol	30.4	7.9 min^{-1}	2.26×10^{7} min^{-1} g_{Pd}^{-1}	This work
PTEB-NFs	0.14 μg	0.1 μmol	76.0	3.3 min^{-1}	2.36×10^{7} min^{-1} g_{Pd}^{-1}	This work
PTEB-NPs	0.375 μg	0.1 μmol	28.4	2.4 min^{-1}	6.40×10^{6} min^{-1} g_{Pd}^{-1}	This work
PTEB-NSs	2.34 μg	0.1 μmol	4.6	2.1 min^{-1}	8.97×10^{5} min^{-1} g_{Pd}^{-1}	This work
Pd@Ru NSs	1.6 μg	0.75 μmol	49.9	0.22 min^{-1}	1.4×10^{5} min^{-1} g_{Pd}^{-1}	[26]
Pd/KCC-1	7.8 μg	0.3 μmol	4.1	0.48 min^{-1}	6.2×10^{4} min^{-1} g_{Pd}^{-1}	[32]
Pd_1/GDY/G	0.13 μg	0.19 μmol	155.5	0.953 min^{-1}	7.3×10^{6} min^{-1} g_{Pd}^{-1}	[33]
Pd/GDYO	1.25 μg	0.067 μmol	5.7	0.322 min^{-1}	2.58×10^{5} min^{-1} g_{Pd}^{-1}	[34]
MpSi-Pd 1	5 μg	1 μmol	21.3	0.159 min^{-1}	3.18×10^{4} min^{-1} g_{Pd}^{-1}	[35]
Pd@RCC3	1.1 μg	0.66 μmol	63.8	1.25 min^{-1}	1.14×10^{6} min^{-1} g_{Pd}^{-1}	[36]
CNT-Pd	29 μg	30 μmol	110.1	0.38 min^{-1}	1.31×10^{4} min^{-1} g_{Pd}^{-1}	[37]
Pd@NC	0.5 μg	0.3 μmol	63.8	0.47 min^{-1}	9.4×10^{5} min^{-1} g_{Pd}^{-1}	[38]
Pd/Fe3O4@SiO2 @KCC-1	2.3 μg	0.3 μmol	13.9	1.1 min^{-1}	4.78×10^{5} min^{-1} g_{Pd}^{-1}	[39]
Pd/tris-trz-PEG	5.3 μg	0.25 μmol	5.0	1.49 min^{-1}	2.81×10^{5} min^{-1} g_{Pd}^{-1}	[40]
Fe@NC@Pd	4.5 μg	30 μmol	709.5	2.4 min^{-1}	5.33×10^{5} min^{-1} g_{Pd}^{-1}	[41]
RGO@C/Pd1	2.92 μg	0.3 μmol	10.9	1.65 min^{-1}	5.65×10^{5} min^{-1} g_{Pd}^{-1}	[42]

References:

(1) Ravel, B.; Newville, M. ATHENA, ARTEMIS, HEPHAESTUS: Data Analysis for X-ray Absorption Spectroscopy Using IFEFFIT. *J. Synchrotron Radiat.* **2005,** *12*, 537–541.

(2) Peng, Z. A.; Peng, X. Mechanisms of the Shape Evolution of CdSe Nanocrystals. *J. Am. Chem. Soc.* **2001,** *123*, 1389–1395.

(3) Liang, L.; Liu, J.; Windisch, J. C. F.; Exarhos, G. J.; Lin, Y. Direct Assembly of Large Arrays of Oriented Conducting Polymer Nanowires. *Angew. Chem. Int. Ed.* **2002,** *41*, 3665–3668.

(4) Liu, J.; Lin, Y.; Liang, L.; Voigt, J. A.; Huber, D. L.; Tian, Z. R.; Coker, E.; McKenzie, B.; McDermott, M. J. Templateless Assembly of Molecularly Aligned Conductive Polymer Nanowires: A New Approach for Oriented Nanostructures. *Chem. Eur. J.* **2003,** *9*, 604–611.

(5) Li, R.; Zhang, X.; Dong, H.; Li, Q.; Shuai, Z.; Hu, W. Gibbs–Curie–Wulff Theorem in Organic Materials: A Case Study on the Relationship between Surface Energy and Crystal Growth. *Adv. Mater.* **2015,** *28*, 1697–1702.

(6) Zhang, T.; Hou, Y.; Dzhagan, V.; Liao, Z.; Chai, G.; Loffler, M.; Olianas, D.; Milani, A.; Xu, S.; Tommasini, M.; et al. Copper-Surface-Mediated Synthesis of Acetylenic Carbon-Rich Nanofibers for Active Metal-Free Photocathodes. *Nat. Commun.* **2018,** *9*, 1140.

(7) Peng, Z. A.; Peng, X. Nearly Monodisperse and Shape-Controlled CdSe Nanocrystals via Alternative Routes: Nucleation and Growth. *J. Am. Chem. Soc.* **2002,**

124, 3343–3353.

(8) Manna, L.; Scher, E. C.; Alivisatos, A. P. Synthesis of Soluble and Processable Rod-, Arrow-, Teardrop-, and Tetrapod-Shaped CdSe Nanocrystals. *J. Am. Chem. Soc.* **2000,** *122*, 12700–12706.

(9) Peng, Z. A.; Peng, X. Formation of High-Quality CdTe, CdSe, and CdS Nanocrystals Using CdO as Precursor. *J. Am. Chem. Soc.* **2001,** *123*, 183–184.

(10) Shi, W.; Luo, Y.; Luo, X.; Chao, L.; Zhang, H.; Wang, J.; Lei, A. Investigation of an Efficient Palladium-Catalyzed C(sp)-C(sp) Cross-Coupling Reaction Using Phosphine-Olefin Ligand: Application and Mechanistic Aspects. *J. Am. Chem. Soc.* **2008,** *130*, 14713–14720.

(11) Mullin, J. W. *Crystallization*; Elsevier Ltd.: Oxford, 2001.

(12) San, X.; Zhao, G.; Wang, G.; Shen, Y.; Meng, D.; Zhang, Y.; Meng, F. Assembly of 3D Flower-Like NiO Hierarchical Architectures by 2D Nanosheets: Synthesis and Their Sensing Properties to Formaldehyde. *RSC Adv.* **2017,** *7*, 3540–3549.

(13) Ji, H.; Zeng, W.; Li, Y. Assembly of 2D Nanosheets into Flower-Like MoO3: New Insight into the Petal Thickness Affect on Gas-Sensing Properties. *Mater. Res. Bull.* **2019,** *118*, 110476.

(14) Wang, S.; Zhang, Z.; Zhang, H.; Rajan, A. G.; Xu, N.; Yang, Y.; Zeng, Y.; Liu, P.; Zhang, X.; Mao, Q.; et al. Reversible Polycondensation-Termination Growth of Covalent-Organic-Framework Spheres, Fibers, and Films. *Matter* **2019,** *1*, 1592–1605.

(15) Williams, V. E.; Swager, T. M. An Improved Synthesis of Poly(p-phenylenebutadiynylene)s. *J. Polym. Sci., Part A: Polym. Chem.* **2000,** *38*, 4669–4676.

(16) Batsanov, A. S.; Collings, J. C.; Fairlamb, I. J. S.; Holland, J. P.; Howard, J. A. K.; Lin, Z.; Marder, T. B.; Parsons, A. C.; Ward, R. M.; Zhu, J. Requirement for an Oxidant in Pd/Cu Co-Catalyzed Terminal Alkyne Homocoupling To Give Symmetrical 1,4-Disubstituted 1,3-Diynes. *J. Org. Chem.* **2005,** *70*, 703–706.

(17) Kresse, G.; Furthmüller, J. Efficient Iterative Schemes for Ab Initio Total-Energy Calculations Using a Plane-Wave Basis Set. *Phys. Rev. B* **1996,** *54*, 11169–11186.

(18) Paier, J.; Hirschl, R.; Marsman, M.; Kresse, G. The Perdew-Burke-Ernzerhof Exchange-Correlation Functional Applied to the G2-1 Test Set Using a Plane-Wave Basis Set. *J. Chem. Phys.* **2005,** *122*, 234102.

(19) Smith, D. G. A.; Burns, L. A.; Patkowski, K.; Sherrill, C. D. Revised Damping Parameters for the D3 Dispersion Correction to Density Functional Theory. *J. Phys. Chem. Lett.* **2016,** *7*, 2197–2203.

(20) Momma, K.; Izumi, F. VESTA 3 for Three-Dimensional Visualization of Crystal, Volumetric and Morphology Data. *J. Appl. Crystallogr.* **2011,** *44*, 1272–1276.

(21) Li, G.; Li, Y.; Liu, H.; Guo, Y.; Li, Y.; Zhu, D. Architecture of Graphdiyne Nanoscale Films. *Chem. Commun.* **2010,** *46*, 3256–3258.

(22) Yang, Z.; Shen, X.; Wang, N.; He, J.; Li, X.; Wang, X.; Hou, Z.; Wang, K.; Gao, J.; Jiu, T.; et al. Graphdiyne Containing Atomically Precise N Atoms for Efficient Anchoring of Lithium Ion. *ACS Appl. Mater. Interfaces* **2018,** *11*, 2608–2617.

(23) He, J.; Wang, N.; Cui, Z.; Du, H.; Fu, L.; Huang, C.; Yang, Z.; Shen, X.; Yi, Y.; Tu, Z.; et al. Hydrogen Substituted Graphdiyne as Carbon-Rich Flexible Electrode for Lithium and Sodium Ion Batteries. *Nat. Commun.* **2017,** *8*, 1172.

(24) Luo, M.; Zhao, Z.; Zhang, Y.; Sun, Y.; Xing, Y.; Lv, F.; Yang, Y.; Zhang, X.; Hwang, S.; Qin, Y.; et al. PdMo Bimetallene for Oxygen Reduction Catalysis. *Nature* **2019,** *574*, 81−85.

(25) Hao, W.; Su, X.; Lu, S.; Wang, J.; Chen, H.; Chen, Q.; Wang, B.; Kong, X.; Jin, C.; Han, G.; et al. Synthesis and Self‐Assembly of Ultrathin Holey Graphdiyne Nanosheets for Oxygen Reduction Reaction. *Small* **2023,** *19*, 2302220.

(26) Zhang, Z.; Liu, Y.; Chen, B.; Gong, Y.; Gu, L.; Fan, Z.; Yang, N.; Lai, Z.; Chen, Y.; Wang, J.; et al. Submonolayered Ru Deposited on Ultrathin Pd Nanosheets Used for Enhanced Catalytic Applications. *Adv. Mater.* **2016,** *28*, 10282−10286.

(27) Jung, A.; Jess, A.; Schubert, T.; Schütz, W. Performance of Carbon Nanomaterial (Nanotubes and Nanofibres) Supported Platinum and Palladium Catalysts for the Hydrogenation of Cinnamaldehyde and of 1-Octyne. *Appl. Catal., A* **2009,** *362*, 95−105.

(28) Chanerika, R.; Shozi, M. L.; Friedrich, H. B. Synthesis and Characterization of Ag/Al2O3 Catalysts for the Hydrogenation of 1-Octyne and the Preferential Hydrogenation of 1-Octyne vs 1-Octene. *ACS Omega* **2022,** *7*, 4026–4040.

(29) Chanerika, R.; Shozi, M. L.; Prato, M.; Friedrich, H. B. The Effect of Coating Pd/Al2O3 and PdAg/Al2O3 Catalysts with [BMIM][DCA] for the Selective Hydrogenation of 1‐Octyne in 1‐Octene. *ChemCatChem* **2023,** *15*, e202201043.

(30) Bruno, J. E.; Dwarica, N. S.; Whittaker, T. N.; Hand, E. R.; Guzman, C. S.; Dasgupta, A.; Chen, Z.; Rioux, R. M.; Chandler, B. D. Supported Ni–Au Colloid Precursors for Active, Selective, and Stable Alkyne Partial Hydrogenation Catalysts. *ACS Catal.* **2020,** *10*, 2565–2580.

(31) Karakhanov, E. A.; Maximov, A. L.; Zolotukhina, A. V.; Yatmanova, N.; Rosenberg, E. Alkyne Hydrogenation Using Pd–Ag Hybrid Nanocatalysts in Surface-Immobilized Dendrimers. *Appl. Organomet. Chem.* **2015,** *29*, 777–784.

(32) Le, X.; Dong, Z.; Li, X.; Zhang, W.; Le, M.; Ma, J. Fibrous Nano-Silica Supported Palladium Nanoparticles: An Efficient Catalyst for the Reduction of 4-Nitrophenol and Hydrodechlorination of 4-Chlorophenol Under Mild Conditions. *Catal. Commun.* **2015,** *59*, 21–25.

(33) Li, J.; Zhong, L.; Tong, L.; Yu, Y.; Liu, Q.; Zhang, S.; Yin, C.; Qiao, L.; Li, S.; Si, R.; et al. Atomic Pd on Graphdiyne/Graphene Heterostructure as Efficient Catalyst for Aromatic Nitroreduction. *Adv. Funct. Mater.* **2019,** *29*, 1905423.

(34) Qi, H.; Yu, P.; Wang, Y.; Han, G.; Liu, H.; Yi, Y.; Li, Y.; Mao, L. Graphdiyne Oxides as Excellent Substrate for Electroless Deposition of Pd Clusters with High Catalytic Activity. *J. Am. Chem. Soc.* **2015,** *137*, 5260–5263.

(35) Kim, T.; Fu, X.; Warther, D.; Sailor, M. J. Size-Controlled Pd Nanoparticle Catalysts Prepared by Galvanic Displacement into a Porous Si-Iron Oxide Nanoparticle Host. *ACS Nano* **2017,** *11*, 2773–2784.

(36) Yang, X.; Sun, J.-K.; Kitta, M.; Pang, H.; Xu, Q. Encapsulating Highly Catalytically Active Metal Nanoclusters Inside Porous Organic Cages. *Nat. Catal.* **2018,** *1*, 214–220.

(37) Kim, J. D.; Choi, M. Y.; Choi, H. C. Catalyst Activity of Carbon Nanotube Supported Pd Catalysts for the Hydrogenation of Nitroarenes. *Mater. Chem. Phys.* **2016,** *173*, 404–411.

(38) Bian, S.-W.; Liu, S.; Guo, M.-X.; Xu, L.-L.; Chang, L. Pd Nanoparticles Partially Embedded in the Inner Wall of Nitrogen-Doped Carbon Hollow Spheres as Nanoreactors for Catalytic Reduction of 4-Nitrophenol. *RSC Adv.* **2015,** *5*, 11913–11916.

(39) Le, X.; Dong, Z.; Liu, Y.; Jin, Z.; Huy, T.-D.; Le, M.; Ma, J. Palladium Nanoparticles Immobilized on Core–Shell Magnetic Fibers as a Highly Efficient and Recyclable Heterogeneous Catalyst for the Reduction of 4-Nitrophenol and Suzuki Coupling Reactions. *J. Mater. Chem. A* **2014,** *2*, 19696–19706.

(40) Wang, C.; Ciganda, R.; Salmon, L.; Gregurec, D.; Irigoyen, J.; Moya, S.; Ruiz, J.; Astruc, D. Highly Efficient Transition Metal Nanoparticle Catalysts in Aqueous Solutions. *Angew. Chem. Int. Ed.* **2016,** *55*, 3091–3095.

(41) Duan, X.; Liu, J.; Hao, J.; Wu, L.; He, B.; Qiu, Y.; Zhang, J.; He, Z.; Xi, J.; Wang, S. Magnetically Recyclable Nanocatalyst with Synergetic Catalytic Effect and Its Application for 4-Nitrophenol Reduction and Suzuki Coupling Reactions. *Carbon* **2018,** *130*, 806–813.

(42) Xi, J.; Sun, H.; Wang, D.; Zhang, Z.; Duan, X.; Xiao, J.; Xiao, F.; Liu, L.; Wang, S. Confined-Interface-Directed Synthesis of Palladium Single-Atom Catalysts on Graphene/Amorphous Carbon. *Appl. Catal., B* **2018,** *225*, 291–297.
